# Nutrition Situation Analysis in the UAE: A Review Study

**DOI:** 10.3390/nu15020363

**Published:** 2023-01-11

**Authors:** Haleama Al Sabbah, Enas A. Assaf, Ayoub Al-Jawaldeh, Afra Salah AlSammach, Haifa Madi, Nouf Khamis Al Ali, Ayesha S. Al Dhaheri, Leila Cheikh Ismail

**Affiliations:** 1 Department of Health Sciences, College of Natural and Health Sciences, Zayed University, Dubai P.O. Box 144534, United Arab Emirates; 2 Faculty of Nursing, Applied Science Private University, Amman 11931, Jordan; 3 World Health Organization Regional Office for the Eastern Mediterranean, Cairo 11516, Egypt; 4 Health Promotion Department, Ministry of Health, Dubai 20224, United Arab Emirates; 5 Department of Nutrition and Health, College of Medicine and Health Sciences, United Arab Emirates University, Al Ain 15551, United Arab Emirates; 6 Department of Clinical Nutrition and Dietetics, College of Health Sciences, University of Sharjah, Sharjah 27272, United Arab Emirates; 7 Nuffield Department of Women’s & Reproductive Health, University of Oxford, Oxford OX3 9DU, UK

**Keywords:** UAE, nutritional status, malnutrition, overweight, obesity, exclusive breastfeeding, stunting, wasting, anemia, food consumption pattern

## Abstract

This review study aimed to assess the nutrition situation in the UAE using published data from 2010 to 2022. It highlights the gaps and challenges that prevail in addressing the nutrition-related problems in the UAE and the opportunities that have been overlooked. The available literature indicates that the UAE is burdened with more than one form of nutrition-related problems, including being underweight, being overweight, obesity, micronutrient deficiencies, and nutrition-related chronic diseases. It is clear that data on micronutrient deficiencies, protein-energy malnutrition, obesity, diabetes, and other nutrition-related diseases among the UAE population are extremely scarce. The UAE has a high prevalence of obesity and diabetes; however, limited studies have been conducted to document this nutritional phenomenon. Few examples of published data are available assessing the burden of stunting, wasting, and being underweight among children under five years of age. Despite the importance of protein-energy malnutrition, no recent publications analyze its prevalence within the UAE population. Therefore, future studies must be conducted, focusing on malnutrition. Based on the literature, and bearing in mind the magnitude of the health issues due to the UAE population’s nutrition negligence, there is an urgent need to assess the population’s nutrient behaviors, to aid policy decision-makers in developing and implementing effective health policies and strategies.

## 1. Introduction

A significant upward trend has been observed in the global burden of non-communicable diseases (NCDs) in the last few decades, with unhealthy eating habits being a chief risk factor [1,2]. According to The Global Burden of Disease project’s 2017 report, the two primary dietary components of this trend—high sodium intake and insufficient dietary fiber—were responsible for a total of six million mortalities worldwide [3]. In addition, the worldwide prevalence of obesity increased by 5.9% [4]. Among the leading causes for the increase in NCDs are the significant lifestyle changes over the past several decades, as a result of which several countries have undergone a nutrition transition [5] that is mainly depicted by an increase in energy, saturated fats, and sugar-dense meals, along with decreased consumption of complex carbohydrates and fibers [6,7].

Ruff et al. [8] reported that since the 1970s, added sugar intake has increased by 50% in the American diet, owing to which the rates of obesity and Type 2 Diabetes Mellites (T2DM) have also grown significantly [9]. A similar trend has been observed in the Eastern Mediterranean countries (EMR) and the Gulf regions. Kuwait, for instance, has undergone a rapid economic and social transition that significantly affected the population’s lifestyle, as indicated by the rising rates of overweight people, obesity, underweight people, and stunting [10]. In Egypt, studies show increasing overweight and obesity rates among children, due to sedentary lifestyles [11]. An increasing rate of obesity and a high prevalence of diabetes and metabolic syndrome have also been reported in the UAE, which can be attributed to the influx of western culture, due to significant economic and cultural development in the last few decades [12,13]. This economic growth attracted expatriates from around the world and increased the variety of food options. Moreover, this has contributed to substantial changes in food consumption habits and behavioral patterns among the UAE’s citizens, increasing their consumption of processed and fatty foods [13].

Abdelaal and his colleagues [14] pointed out that early-life obesity served as a lifelong determinant of morbidity and chronic diseases such as metabolic disorders, cardiovascular diseases, and cancers. According to Al-Haddad et al., the Middle East has poor eating habits, with a high intake of unhealthy calories and a low intake of micronutrients [15]. Moreover, studies conducted in the Middle East have demonstrated that preschoolers show suboptimal health and food intake, with reports of several deficiencies and overnutrition [16]. Data from a recent national cross-sectional study conducted in Kuwait indicated a 28.4% prevalence of obesity in school-aged children (5–19 years) in 2019, which is among the highest rates in the Eastern Mediterranean region [17]. Similarly, high rates of overnutrition have been reported in the UAE, with a prevalence of overweight and obesity among school-aged children reaching 34% and 18.9%, respectively, suggesting that the UAE has one of the highest national rates of childhood obesity worldwide [15]. Apart from obesity, undernutrition also persists as an issue affecting children, due to unhealthy eating patterns [18].

A study of school-age children in Egypt reported that 8% were underweight and 36% were diagnosed with anemia [19]. Another study, of cerebral palsy children in Saudi Arabia, found that more than half of the children were malnourished, and several children were undernourished and stunted [20]. Similarly, 7.6% of Emirati schoolchildren were found to be underweight in the United Arab Emirates [21]. Based on national data for underweight, stunted, and overweight/obese children, the rates were reported in a fact sheet from a global school-based survey in the Emirates as 3.8%, 39.1%, and 16.2%, respectively [22]. For adults, the data indicate a prevalence of overweight and obesity of 74% and 37.2%, respectively [23]. Regarding anemia, available data indicates a prevalence rate of 26% and 24.3% in women of reproductive age and pre-schoolchildren, respectively [24,25,26], which are considered to be moderate public health problems, according to the WHO’s classifications [27].

The United Arab Emirates is a country consisting of a multicultural population and, hence, it has a diverse food-abundant environment. The availability of multiple fast-food chains and easily accessible calorie-dense meals, along with several other factors, have contributed to the deteriorating health of the population. The decline in health is evidenced by rising rates of obesity, overweight, and nutrition-related NCDs. The UAE has a high prevalence of obesity in adolescents and adults; however, data are scarce. There have been limited studies conducted to document this nutritional phenomenon. Bearing in mind the magnitude of the health issues due to nutrition negligence within the population, there is an urgent need to assess nutrient intake to assist policy decision-makers in developing and implementing effective health policies and strategies in alignment with the vision of the UAE government to improve the population’s health and reduce healthcare costs. Therefore, this study aims to assess the nutrition situation in the UAE by reviewing the available literature.

## 2. Materials and Methods

The UAE has 9,282,410 inhabitants based on the most recent national statistics in 2020 [28], with a female-to-male ratio of 2:1. Total life expectancy at birth is 79.7 years; for males, life expectancy at birth is 78 years, and for females the figure is 81.4 years, as reported in 2020 [28]. About 85% of the UAE’s inhabitants were less than 14 years old, and the mortality rate of children under five years was 5.1 per 1000 live births, as reported in 2020 [29]. Concerning food insecurity, data in 2020 indicated a prevalence of 0.8% for severe food insecurity [30].

To assess the nutritional situation in the UAE, a systematic database search was carried out from January 2022 to November 2022 to identify relevant studies. Only studies published in the English language were included in this review. The scientific databases included in the search included PubMed, Science Direct, Google Scholar, ResearchGate, Scopus, the UAE Ministry of Health’s website, the Ministry of Health and Prevention strategic and action plan 2017–2021 [31], the WHO databases, the Global School-Based Student Health Survey [32], the WHO/UNICEF Joint child malunion estimate 2021 [33], the STEP wise Approach to NCD Risk Factor Surveillance (STEPS) 2021 [34], and the WHO/Nutrition Country Profile [25]. The trends and prevalence of various nutritional indicators are shown to be present when there is available national data over the years. Specific national indicators are evaluated based on the WHO target goals for 2025 [35]. The search terms used included “under-nutrition”, “obesity”, “stunting”, “malnutrition”, “micronutrient deficiency”, “nutrition status”, “diet-related risk factors”, “nutrition government policy”, “national nutrition strategy”, or “nutrition health policy”, in combination with “United Arab Emirates” or “UAE”.

**Figure 1:**
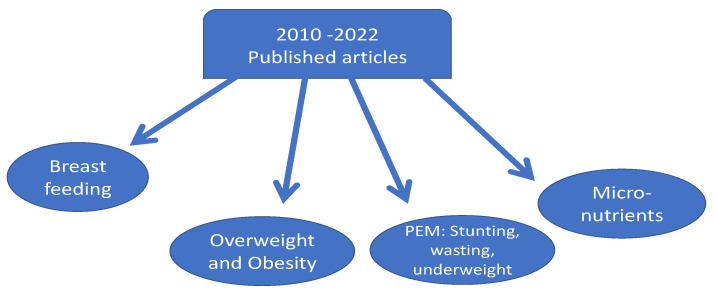
Literature review main themes.

The search was further filtered and limited to articles published from 2010 to 2022. Selected sources included journals, books, book chapters, and government data sets. Unreliable sources, such as magazines and newspaper articles, were not included in this review. A total of 48 articles were extracted using the above filters and keywords. After reviewing all relevant literature, the following four main themes were established (Figure 1):

1. Exclusive breastfeeding, complementary feeding, and low birth weight;
2. Protein-energy malnutrition (stunting, wasting, and underweight);
3. Obesity and overweight by age groups;
4. Micronutrient deficiency.

## 3. Results

### 3.1. Exclusive Breastfeeding, Complementary Feeding, and Low Birthweight

Studies have shown that exclusive breastfeeding has a positive effect on preventing childhood obesity. A study by Altarrah [36] indicated that a longer duration of breastfeeding, followed by the introduction of complementary foods, was associated with a lower BMI z-score. However, the prevalence of exclusive breastfeeding in infants up to 6 months of age in the UAE was considerably lower (59.7%), as indicated in the National Health Survey 2018 and rates of 44.3% in the capital city of Abu Dhabi, 22% in Dubai, 31.1% in Sharjah, and 46.7% in the Northern Emirates of the UAE [37,38,39,40].

Regarding complementary feeding, a study conducted in three Emirates (Abu Dhabi, Dubai, and Al Ain) found that 83.5% of infants received solid food before reaching six months of age [41]. The reasons for early initiation of feeding, as shown in the study, included breastfeeding termination, new pregnancy, insufficient milk supply, and situations in which infants weaned themselves [41]. Another study conducted in Abu Dhabi found that 36.2% of children who were ≥6 months of age had a minimum acceptable diet, whereas 47.3% of children who were not breastfed met the requirements for minimum meal frequency; children who were 6–23 months old were the least compliant (21.9%). On the other hand, many children were fed sugar-containing snack items [42]. 

**Figure 2:**
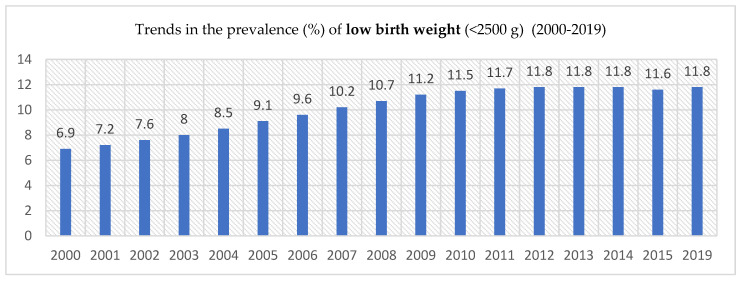
Trends in the prevalence (%) of low birth weight (<2500 g) in the UAE (2000–2019) [29,44].

Another population-based study, conducted in 2022 in Sharjah, Dubai, and Abu Dhabi, found that 98% of children had a timely introduction to complementary feeding. However, it was reported that toddlers aged 12–23.9 months did not meet food groups’ recommendations, as follows: 93% did not meet the recommendations for vegetables; 54% did not meet the recommendations for lean meat and beans; 87% did not meet the recommendations for fruits; 48% did not meet the recommendations for milk/dairy; and 33% did not meet the recommendations for grains [43]. Low birth weight prevalence, as reported by UNICEF, showed an increase between 2000 and 2015; the prevalence rate in 2015 (11.6%) was nearly twice as high as the rate in 2000 (6.9%) (Figure 2) [44]. In comparison, based on the UAE Ministry of Health’s recent report, the prevalence of low birth weight reported in 2019 was 11.8% [29]. In addition, a study conducted in Abu Dhabi found that the prevalence of low birth weight was 9.4% in 2020 [45].

### 3.2. Protein Energy Malnutrition (Stunting, Wasting, and Underweight) among Children under 5 Years

Karavetian et al. [46], in their cross-sectional study, aimed to determine the prevalence of malnutrition among 70 hemodialyses (HD) patients in a tertiary hospital-based HD unit in the UAE. The findings revealed that almost half of the sample was malnourished, according to the malnutrition–inflammation score (MIS) (48.57%) and the Global Leadership Initiative on Malnutrition (GLIM) criteria (54.29%). In addition, Karavetian et al. [47] found in another cross-sectional multicenter study in the UAE that the prevalence of malnutrition among hospitalized patients was 27.7%, based on the Global Leadership Initiative on Malnutrition (GLIM) (Table 1). One study of toddlers between zero and 23.9 months of age, conducted in 2020 by Cheikh Ismail et al. in Abu Dhabi, Dubai, and Sharjah, found that among the sampled children, 15% were stunted, 4% were malnourished, and 7% were overweight/obese, while 18% were at risk of being overweight. In addition, 98% of infants had started complementary feeding on time [43].

The results of a recent population-based study of infants up to 24 months old, conducted in three Emirates in the UAE (Sharjah, Dubai, and Abu Dhabi), indicated that 8% were wasted, 15% were stunted, 4% were malnourished, 18% were at risk of being overweight, and 7% were overweight/obese [43]. Another recent study of children between 0 and 4 years of age, conducted in the three Emirates of Dubai, Abu Dhabi, and Sharjah, found that 17% were at risk of being overweight, 5% were overweight, 3% were obese, and 6% were wasted, while no children were found to be stunted [48] (Table 1).

### 3.3. Obesity and Overweight by Age Groups

#### 3.3.1. Children and Adolescents; Childhood

Obesity is a global epidemic and a lifelong determinant of morbidity and chronic diseases such as metabolic disorders, cardiovascular diseases, and cancers [49]. The national survey of obesity among school-age children (5–17 years old) in the UAE in 2019 reported that 17.35% were obese, with the highest percentage in Fujairah Emirate and the lowest in Dubai (20.13 and 15.59, respectively) (Supplementary File S1), rendering it a public health priority. Despite its significance, only 10 studies conducted in the UAE reported data on childhood and adolescent obesity (Table 2). Data from these studies pointed out several factors leading to early obesity, such as socioeconomic, geographical, and environmental factors. However, the predominant factors were found to be individual and parental behaviors [50]. Another study of adolescent schoolchildren, conducted in Al Ain city, found that the prevalence of metabolic syndrome increased with obesity in children and adolescents [51]. Studies have emphasized the positive impact of exclusive breastfeeding on preventing childhood obesity.

**Figure 3:**
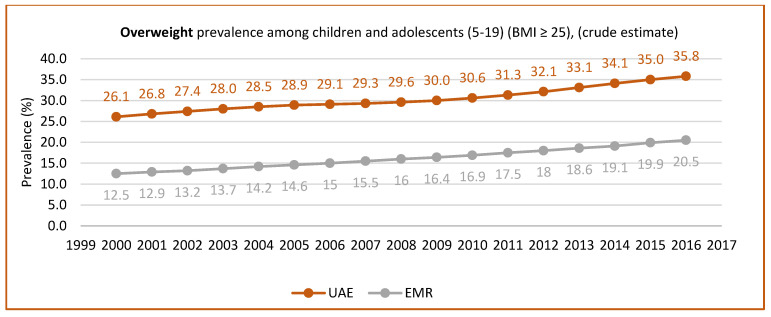
Overweight prevalence in children and adolescents (aged 5–19 years) (BMI ≥ 25) (crude estimate). Source: https://www.who.int/data/gho/data, http://www.healthdata.org/results/country-profile (accessed on 5 December 2022) [33,35].

**Figure 4:**
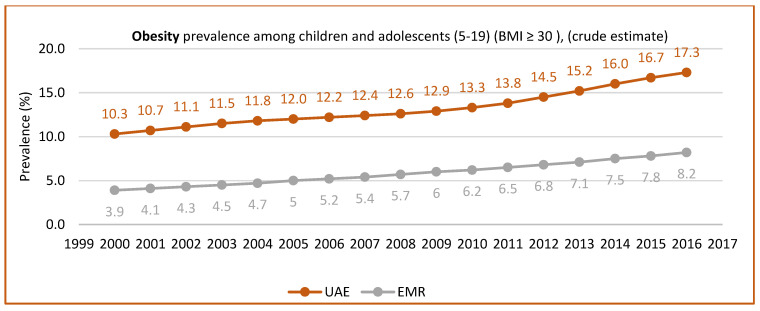
Obesity prevalence in children and adolescents (aged 5–19 years) (BMI ≥ 30) (crude estimate). Source: https://www.who.int/data/gho/data. http://www.healthdata.org/results/country-profile (accessed on 5 December 2022) [33,35].

Abduelkarem et al. [52] reported a significantly high percentage of children engaging in fast food consumption and physical inactivity. They also found that the type of food, age, and time spent watching TV were significantly associated with BMI. In addition to the role of lifestyle, a few studies pointed out the role of gender, wherein males were more prone to being obese than females. Baniissa et al. [53] found that predictors of obesity based on BMI were mainly the following: consuming less than five servings of fruits/vegetables per day, being physically inactive, and being male. Similarly, AlBlooshi et al. [54] confirmed a steady rise in obesity in children between 3 to 18 years of age, especially in boys. They reported that 10.3% of boys and 3.0% of girls were very obese between the ages of 15 to 18 years. However, another study found that the adverse effects of distance learning during the lockdown in the UAE included unhealthy food consumption and increased snacking, which might affect children’s weight [55]. Figure 3 and Figure 4 show the prevalence of overweight and obesity in children and adolescents aged between 5 to 19 years, according to the UAE country profile.

**Table 2:** Overweight and Obesity among Children.

	Year	Title	Settings	Sample Size	Author
1	2013	Parental Weight Perceptions: A Cause for Concern in the Prevention and Management of Childhood Obesity in the United Arab Emirates.	Students and their parents from public schools (grades 1–12) Abu Dhabi	945 Emirati students and their parents.	Aljunaibi et al. [56]
2	2016	Increasing obesity rates in school children in United Arab Emirates.	Governmental schools in Ras Al-Khaimah.	44,942 students	AlBlooshi et al [54].
3	2018	PO596 Emergence of CVD Risk Factors in Elementary School Children in United Arab Emirates: Role of Obesity?	114 schools in Al Ain, UAE	1186 participants	Shehzad et al [57].
4	2018	Preschool obesity in the United Arab Emirates: determinants and effectiveness of the Ten Step Healthy Lifestyle Tool for Toddlers: Eat Right Emirates study	Private school and Emirates National Schools (ENS) in the Al Ain, UAE	402 preschool-aged children between 2 and 6 years	Altarrah [36]
5	2018	Content analysis of media coverage of childhood obesity topics in UAE newspapers and popular social media platforms, 2014–2017.	Review study on related to childhood obesity usingGulf News (The National and Al Ittihad) and social media posts.	-152 newspaper articles, -57 Facebook posts, -50 Twitter posts, -14 posted YouTube videos, and -13 media releases.	Awofeso et al [50].
6	2019	Dyslipidemia, subclinical inflammation, hepatic cholestasis and endothelial dysfunction in schoolchildren with excess fat: A study from the United Arab Emirates.	25 Alain governmental schools grades 2, 6, 10	967 Emirati students, aged 8, 12 and 16 year	Aburawi et al [20].
7	2020	Metabolic syndrome among children aged 6 to 11 years, Al Ain, United Arab Emirates: Role of obesity	114 schools in Al Ain, Abu Dhabi, UAE	1186 participants	Shah et al [51].
8	2020	Prevalence of overweight/obesity, anaemia and their associations among female university students in Dubai, United Arab Emirates: A cross-sectional study.	National universities in the UAE	300 students	Al Sabbah [58]
9	2020	Prevalence and determinants of overweight/obesity among school-aged adolescents in the United Arab Emirates: a cross-sectional study of private and public schools.	Private and public secondary schools	Adolescents (13–19 years) -434 from private schools -498 from public schools	Baniissa et al [53].
10	2020	Obesity and its associated risk factors among school-aged children in Sharjah, UAE	8 schools in Sharjah.	Students aged 6–11 years 678 (299 boys and379 girls)	Abduelkarem et al [52].

#### 3.3.2. Adults

Limited studies have been conducted in the UAE regarding the nutritional status of adults in the past decade. A total of 23 articles were extracted, reporting data on obesity and overweight in adults (Table 3). These studies primarily reported data on the prevalence rates, the risk factors, and the effects of obesity. A systematic review by Radwan et al. [59] reported high prevalence rates of obesity, diabetes, hypertension, and metabolic syndrome in all studies. Similar findings were reported in other studies as well. Razzak et al. [60], in their cross-sectional study, found that the prevalence of obesity was higher in proportion among adult females than in adult males in UAE.

Extensive evidence points to the role of obesity in the development of several chronic diseases. Sulaiman et al. [61] conducted a study of 3064 expatriates in the UAE. Their findings revealed a high prevalence of overweight and obesity in the sample. In addition, the prevalence rates for chronic disorders, such as diabetes, hypertension, and hypercholesterolemia, were 15.5%, 31.8%, and 51.7%, respectively, with each disorder being higher in those people with obesity. Another study, conducted on students at Ajman University, observed a correlation between high BMI and prehypertension, as well as high glucose levels and other risk factors such as smoking and a sedentary lifestyle [62]. Shah and his colleagues conducted a study of male South Asian immigrants in the UAE to assess the relationship between obesity and cardiac diseases. Their findings revealed that the overall prevalence of BMI-derived obesity rate was 44.7%, and the central obesity rate in males was 66.7%. The results also indicated that 30% and 9% of the individuals were diagnosed with hypertension and diabetes, respectively [63].

A study conducted by Shehzad, Shah, Aziz, and Al-Maskari [57] reported that participants who were overweight or obese were more likely to have elevated cardiometabolic risk factors, irrespective of age and gender. Moreover, Al Sharbatti et al. [64] indicated that the risk of hypertension increased by 4.3 times for participants who had general obesity or abdominal obesity. Findings from a population-based study conducted by Mahmoud and Sulaiman [65] showed that the prevalence of obesity was 66.4% based on BMI, 61.7% based on waist circumference, 64.6% based on waist–hip ratio, and 71% based on neck circumference. They observed that obesity based on BMI, waist circumference, and waist–hip ratio was significantly associated with cardiometabolic risks. Similar results were reported by Abdullah, Al-Junaibi, and Nagelkerke [66], who concluded that high BP was strongly related to increased body weight and BMI.

Other studies highlighted several leading causes of obesity in the population. A study by Ng et al., describing the nutrition transition in the UAE [13], found that 43% of girls and 38% of boys consumed more calories than their energy requirements. Moreover, the data revealed that snacking, consumption of caloric beverages, and lack of physical activity, especially among females, are the factors that represent a potential risk of cardiometabolic problems in the UAE. Another study reported a positive relationship between obesity and eating outside the home, income, and computer use [67]. Vats et al. [68] conducted a wellness survey in which participants reported that being obese affected their daily activities negatively. Poor education, time restrictions, low motivation, and other undefined factors were the cause of not taking measures to reduce weight. Moreover, it was found in the same study that for 72% of healthcare professionals, the main causes of obesity were a sedentary lifestyle, lack of awareness, high caloric diet, cultural/genetic predisposition, and adverse climatic conditions. More recently, factors related to the COVID-19 lockdown, which has induced physical inactivity, were found to be related to increased BMI in 10 Arab countries, including UAE [69]. Another study by Cheikh Ismail et al. showed that almost one in three people in the UAE reported weight gain during the COVID-19 pandemic, concurrently with reports of unhealthy eating patterns, lower physical activity levels, and increased screen time [70]. In addition, a follow-up study in four Arab countries, including the UAE, indicated that several unfavorable lifestyle and dietary habits persisted even after the availability of the COVID-19 vaccine, indicating that people might have continued with eating habits acquired during the COVID-19 lockdown [71]. One recent study found that smoking was significantly associated with high BMI in university students in both Dubai and Palestine [72].

**Figure 5:**
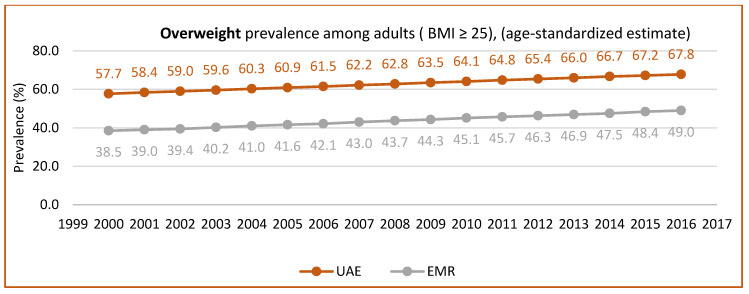
The prevalence of overweight in adults (BMI ≥ 25) (age-standardized estimate), the UAE trend (1999–2017). Source: https://www.who.int/data/gho/data, http://www.healthdata.org/results/country-profile (accessed on 5 December 2022) [33,35].

**Figure 6:**
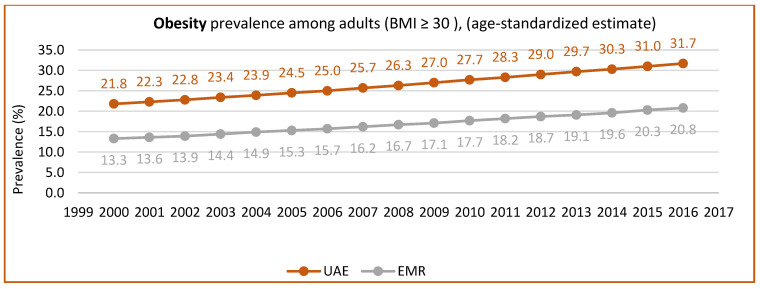
The prevalence of obesity in adults (BMI ≥ 30) (age-standardized estimate) UAE trend (1999–2017). Source: https://www.who.int/data/gho/data, http://www.healthdata.org/results/country-profile (accessed on 5 December 2022) [33,35].

**Figure 7:**
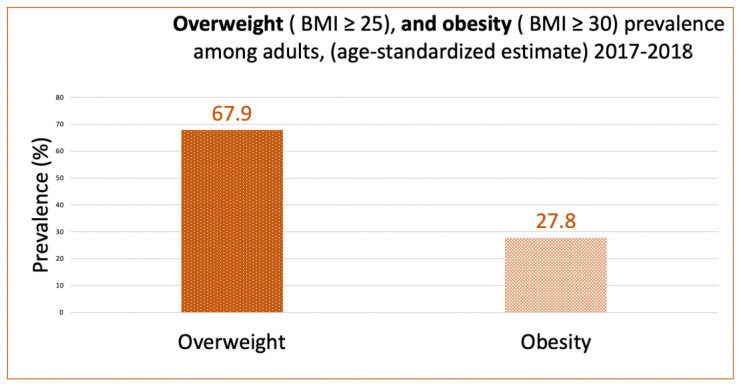
The prevalence of overweight and obesity (age-standardized estimate), 2017–2018 (the UAE). Source: https://www.mohap.gov.ae/Files/MOH_OpenData/1556/UAE_NHS_2018.pdf (accesséd on 5 December 2022) [82].

Figure 5 and Figure 6 show the prevalence of overweight and obesity among adults in the UAE from 1999 to 2017, according to the UAE country profile, while Figure 7 shows data from 2017 to 2018, according to the UAE Ministry of Health. These figures clearly show the increase in overweight and obesity over the years.

**Table 3:** Overweight and Obesity in adults.

	Year	Title	Settings	Sample Size	Author
1	2011	The Use of Obesity Indicators for the Prediction of Hypertension Risk among Youth in the United Arab Emirates.	Gulf Medical University of Ajman, UAE.	110 first year students	Al-Sharbatti et al. [64]
2	2011	Nutrition transition in the United Arab Emirates.	Households in all seven emirates	628 households in all seven emirates	Ng et al. [13]
3	2013	Obesity hypoventilation syndrome in obstructive sleep apnea patients in the United Arab Emirates: a retrospective cross-sectional study.	Respiratory Care Unit and Sleep Disorder Centre of the Zayed Military Hospital United Arab Emirates.	212 adult patients	Alzaabi et al. [73]
4	2013	What determines obesity in oil-rich UAE? New evidence from survey data.	University undergraduate students	721 students	Katsaiti and El Anshasy [67]
5	2014	High blood pressure and its association with body weight among children and adolescents in the United Arab Emirates.	School in Abu Dhabi, UAE.	999 Emirati national school students aged 6–17 year	Abdulle et al. [66]
6	2015	Association between acculturation, obesity and cardiovascular risk factors among male South Asian migrants in the United Arab Emirates—A cross-sectional study.	Visa health screening center in Abu Dhabi (UAE	1375 males	Shah et al. [63]
7	2016	Laparoscopic Sleeve Gastrectomy for Morbid Obesity: UAE Tertiary Care Hospital Initial Experience.	Various clinics, UAE	109 Morbidly obese patients	Gondal et al. [74]
8	2016	Subclinical inflammation and endothelial dysfunction in young patients with diabetes: A study from United Arab Emirates.	-Three diabetes centers in Abu Dhabi. -Public schools -UAE University	-79 with type 1 diabetes -55 patients with type 2 diabetes -47 controls.	Aburawi et al [75].
9	2017	The prevalence and risk factors of obesity in the United Arab Emirates.	Systematic review using: PubMed, Scopus, Science Direct database, and other local journals	Systematic review	Razzak et al. [60]
10	2017	Obesity and Sleep-Related Breathing Disorders in Middle East and UAE.	Systematic review Middle East (ME) and United Arab Emirates (UAE)	Systematic review	Vats et al. [68]
11	2017	Prevalence of overweight and obesity in United Arab Emirates Expatriates: The UAE National Diabetes and Lifestyle Study	Adult ≥ 18 years expatriates who had resided in the UAE for at least 4 years.	2724 participants (2204 Males and 520 Females)	Sulaiman et al. [61]
12	2018	The epidemiology and economic burden of obesity and related cardiometabolic disorders in the United Arab Emirates: A systematic review and qualitative synthesis.	Systematic review using MEDLINE, PubMed, Embase, Cumulative Index to Nursing and Allied Health Literature (CINAHL), Index Medicus.	Systematic review	Radwan et al. [59]
13	2018	Clinical Practice Recommendations for the Management of Obesity in the United Arab Emirates.	A multi-disciplinary panel of experts who treat patients with overweight and obesity reviewed and streamlined international recommendations.	A multi-disciplinary panel of experts	Abusnana et al. [76]
14	2018	Multiple genetic variations confer risks for obesity and type 2 diabetes mellitus in Arab descendants from UAE.	United Arab Emirates	Tested 87, 58, and 586 SNPs in a previous genome-wide significance level for associations with BMI (n = 880), WC (n = 455), and height (n = 897)	Osman et al. [77]
15	2019	Prevalence Of Diabetes, Hypertension And Obesity And Associated Factors Among Students Of Ajman University, United Arab Emirates.	Ajman University, UAE	250 students	Shahwan et al [62].
16	2019	Relationship of salivary adipocytokines, diet quality, physical activity, and nutrition status in adult Emirati females in United Arab Emirates.	University of Sharjah	90 normal-weight, overweight, and obese adult females	Attlee et al. [78]
17	2019	Prevalence of obesity among adults in Ras Al Khaimah, United Arab Emirates.	Ras Al Khaimah, UAE	544 adults	Kalavathy et al. [79]
18	2019	Added sugar: Nutritional knowledge and consumption pattern of a principal driver of obesity and diabetes among undergraduates in UAE.	University undergraduate students	400 undergraduate students from UAE	Khawaja et al. [6]
19	2020	Implication of genetic variants in overweight and obesity susceptibility among the young Arab population of the United Arab Emirates.	Khalifa and Zayed Universities in Abu Dhabi	392 controls and 318 overweight/obese young Emiratis (aged 18–35 years).	El Hajj Chehadeh et al. [80]
20	2020	An integrative phenotype–genotype approach using phenotypic characteristics from the UAE national diabetes study identifies HSD17B12 as a candidate gene for obesity and type 2 diabetes.	UAE nationals and expatriates living in Sharjah, Dubai, and the Northern Emirates for at least four years.	708 chromosomal regions associated with UAEDIAB-phenotypes.	Hachim et al. [81]
21	2021	Significance and agreement between obesity anthropometric measurements and indices in adults: a population-based study from the United Arab Emirates.	Sharjah, Ajman, Ras al-Khaimah, Fujairah, and Umm al-Quwain.	3531 participants.	Mahmoud and Sulaiman [65]
22	2022	The Impact of COVID-19 on Physical (In)Activity Behavior in 10 Arab Countries	online survey in 10 Arab countries including UAE	A total of 12,433 participants.	Al Sabbah et al. [69]
23	2022	Prevalence of smoking (cigarette and waterpipe) and its association with obesity/overweight in UAE and Palestine	10 largest universities in west bank Palestine and Dubai	3800 university students (1900 Dubai and 1900 West Bank)	Al Sabbah et al. [72]
24	2022	Eating Habits and Lifestyle during COVID-19 Lockdown in the United Arab Emirates: A Cross-Sectional Study	Online questionnaire United Arab Emirates	1012 participants	Cheikh Ismail et al. [71]
25	2022	Assessment of Dietary and Lifestyle Responses After COVID-19 Vaccine Availability in Selected Arab Countries	Online questionnaire used in United Arab Emirates, Lebanon, Palestine territories, and Jordan	2259 participants	Cheikh Ismail et al. [71]

### 3.4. Micronutrient Deficiency

Sadiya et al. [83] reported on vitamin D status in the UAE population and its effect on metabolic parameters in individuals with obesity and type 2 diabetes (T2D). The sample consisted of 309 males and females randomly selected from the Rashid Center for Diabetes and Research, a tertiary diabetes care facility in the Emirate of Ajman in the UAE. The key finding of their study was the high prevalence (83.2%) of vitamin D deficiency in the sample of the Emirati population with obesity and T2D. However, an RCT done by Sadiya et al. [84] found that no significant impact of vitamin D supplementation on body weight, fat mass, or waist circumference in T2D obese vitamin-D-deficient participants was observed, despite one year of a high daily dose of vitamin D3 supplementation. A study conducted in Abu Dhabi Ain found that the prevalence of vitamin D deficiency was very high (79.6%) [85].

No progress was reported toward achieving the target of reducing anemia in women of reproductive age, with 24.3% of women aged 15 to 49 years affected [26]. Two studies indicated the prevalence of iron deficiency anemia in children under five years of age. The first study, conducted in 2019 with 259 infants in the Hatta suburb in the UAE, found that 22% of the infants were anemic [86], while the second study conducted by Faysial et al. found that the prevalence of anemia was 31% of hospitalized cases that were diagnosed as iron deficiency anemia in Dubai [87].

**Figure 8:**
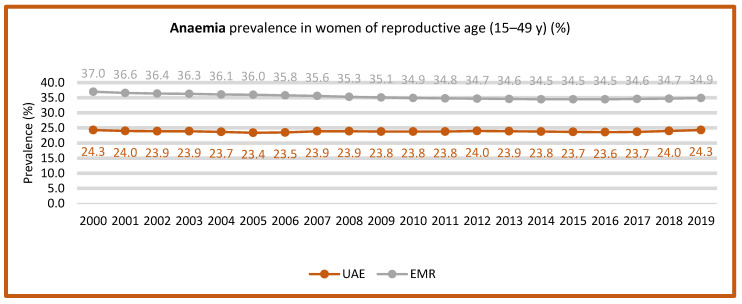
Trends of iron deficiency anemia prevalence among women of reproductive age (15–49 years of age) (2000–2019). Source: https://www.who.int/data/gho/data/themes/topics/anaemia_in_women_and_children (accessed on 5 December 2022) [26].

A cross-sectional study conducted by Al Sabbah [58], using a sample of 300 university students selected via systematic random sampling, found a significant association between anemia and total body fat, wherein obese and underweight students were found to be two times more likely to have anemia in comparison to normal-weight students (Table 4). Figure 8 shows that the prevalence of iron deficiency anemia in women of reproductive age (15–49) was almost stable from 2000 to 2019.

## 4. Discussion

### 4.1. Exclusive Breastfeeding, Complementary, and Low Birthweight

Exclusive breastfeeding in the UAE was discussed in two studies [38,39], and both indicated a lack of adherence to WHO recommendations. Factors contributing to low or no adherence to exclusive breastfeeding, including duration of breastfeeding, the skin-to-skin period, being underweight, the last infant’s sex, having a maid at home, the number of children, the living place, and working mothers, were found in other global studies [88,89,90]. Studies showed that early nutrition might be an important factor in improving physical growth and cognitive development, enhancing immunity, and preventing non-communicable diseases [91,92]. Other barriers to adequate breastfeeding and mixed feeding, as discussed in global studies, might be the lack of designated breastfeeding facilities in the working place or shopping malls, community attitudes toward breastfeeding, and formula feeding as enhanced by milk companies and the market [42,92,93,94].

Complementary feeding was found to be timely initiated. However, factors associated with complementary feeding and not meeting the recommendations of nutritional groups for the early introduction of complementary feeding for infants and toddlers were not well discussed, requiring further attention. One study conducted for other countries of the MENA region (Jordan, Lebanon, Palestine, Sudan, and Egypt) found that maternal education, paternal/maternal age, social status, cultural factors and settings, and the utilization of healthcare services were all associated with minimum meal frequency, minimum dietary diversity, and minimum acceptable diets [95]. Interestingly, studies have shown that mothers who adhere to breastfeeding practices were working on dietary diversity practices for their infants and toddlers, with less sugar and less fatty diets [95,96].

Low birth weight was shown to be decreased but not significant in the UAE. However, the factors associated with low birth weight were discussed in a study by Taha and her colleagues [45], including cesarean-section delivery, first-child order, and preterm birth. These results were in agreement with other studies in Nepal and Ethiopia concerning comorbidity and low iron intake during pregnancy [97,98].

### 4.2. Protein Energy Malnutrition (Stunting, Wasting, Underweight) in Children under 5 Years of Age

In the UAE, seven studies discussed malnutrition (stunting, wasting, and being underweight). Those seven studies suggested and recommended a balanced diet with more focus on fruits and vegetables and less intake of sugar, fatty diets, and sweet beverages. Nonetheless, as stated in the literature review, in addition to obesity, undernutrition also remains an issue. Studies in Saudi Arabia and Egypt reported several children to be undernourished and stunted [18,19,99]. Similarly, a study conducted in the UAE in 2013 reported that 7.6% of Emirati schoolchildren were underweight [21]. One study published in 2022 found that the prevalence rate in three Emirates in the UAE showed undernutrition, stunting, and wasting. However, because undernutrition was not the primary aim of that study, the related factors were not discussed. A study in India indicated that per capita income did not influence child nutrition; instead, proper healthcare services, proper health follow-up, attitudes toward the overall health status of children under five years of age, and environmental aspects were the influencing factors [100]. According to a population-based study by Cheikh et al. in 2022 [43], the prevalence of wasting was 8%, the prevalence of stunting was 15%, the risk of being overweight was 18%, and the prevalence of obesity was 7%. The figure for stunting was less than the global level of stunting in 2020 data (22%); the figures for wasting and being overweight in the global data were 6.7% and 5.7%, respectively [101]. These reported prevalence rates for the UAE can provide us with an indication of the real problems. The results in low- and middle-income countries showed several factors that can play a role in stunting and wasting for children under five years of age. These factors included low maternal body index, short paternal height, poor paternal health status, and socioeconomic factors, in addition to parental educational level [102].

### 4.3. Obesity and Overweight

#### 4.3.1. Obesity in Children

Studies concerning obesity among children showed an increasing prevalence rate in the UAE, exceeding the global prevalence rate of 5.7% in 2020 [101]. It is important to note that childhood obesity plays a role in determining the future health of children and the development of chronic diseases. Aburawi et al. [75] reported that children with excess fat had increased risks of developing systemic inflammation, dyslipidemia, endothelial dysfunction, cholestasis, and diabetes. Obesity among children is considered one of the public health risks and problems, as it may continue into adulthood and lead to various psychological effects as well as physical complications [103]. Factors that were found to be associated with childhood obesity in the UAE and which were consistent with those in other studies include the following: engaging in fast food consumption and sugar-sweetened beverages [104,105], physical inactivity [106], age (as more obesity is found in adolescence than in younger-aged children) [107], time spent on TV and other screens [108,109], and gender (as boys were more prone to being obese than girls) [107]. Due to globalization and the diversified population of the UAE, the nutrition culture in the UAE has been influenced by international cuisines, and this has had an impact on every aspect of local food culture.

#### 4.3.2. Adult Obesity

The UAE ranks as the fifth most obese country in the world, with increasing cardio-metabolic risks [78], as it exceeds the global level (13.2%) [110]. It is crucial to address the increasing prevalence of obesity, as it has negative impacts and puts significant strain on the healthcare system. Factors associated with overweight and obesity among adults in the UAE include the following: snacking, consumption of caloric beverages, lack of physical activity, lifestyle changes (such as decreased intake of fruits and vegetables), and smoking. These factors were consistent with the factors identified in studies in other countries, regionally and globally [105,111,112,113,114]. Moreover, the prevalence of obesity and overweight was higher among females in the UAE, which could be explained by the fact that women have more cultural constraints in their lifestyles and physical activities than men [115]. Smoking and physical activity were associated with BMI in the UAE, as the smoking of waterpipes was associated with increased waist circumference and increased BMI [72]. Moreover, TV viewing and reduced physical inactivity were associated with increased BMI among adults in the UAE [69]. In addition, one study found that stress was associated with obesity in diabetic obese patients in the UAE [116].

### 4.4. Micronutrients

Few studies have reported information on vitamin D deficiency and iron deficiency anemia in the UAE. However, the role of several other vitamins and minerals is also crucial in achieving a well-nourished body. In the Global Burden of Disease project, iron deficiency anemia (IDA) was ranked number nine among the 26 modifiable risk factors for death [117]. In 2019, the prevalence of iron deficiency anemia in women of reproductive age (24.3%) was close to that of the global rate (29.9%) [26], while the two studies showed a high number of children under the age of 5 with iron deficiency anemia. The prevalence of iron deficiency anemia in children was similar to that in Saudi Arabia (51%) [100]. IDA among children must be treated with special attention because it is associated with cognitive abilities, learning abilities, and motor functions [118,119].

## 5. Conclusions and Recommendations

As indicated by the literature, very limited studies have been conducted in the United Arab Emirates to document nutrition-related problems. Further research is urgently required to fully evaluate nutritional behaviors in light of the severity of the health problems caused by nutritional neglect. In addition, it is important to increase nutrition awareness about healthy food choices within the population, along with developing interventions aimed at modifying the health policies in the country, to remain in alignment with the vision of the UAE government to improve the population’s health and to reduce health care costs. Future studies must be conducted to bridge the gaps in the literature and to further aid the policy decision-makers in developing and implementing effective nutrition policies and strategies to improve the population’s health and quality of life.

The prevalence of wasting, stunting, overweight, and obesity in the UAE was less than that of the global level for stunting and more than that of the global level for wasting and overweight. The results from the UAE indicated real problems and that more studies are required to measure the factors behind these results. Few published studies have analyzed protein-energy malnutrition in the UAE population. Therefore, further attention and more focus are required to investigate the associated factors behind this issue in the UAE. Obesity among children in the UAE is prevalent and associated with fast food consumption, sugar-sweetened beverages, physical inactivity, and time spent on TV and other screens.

There is an urgent need for more studies about obesity and overweight in the UAE population. Childhood obesity is considered a public health problem, as it may continue into adulthood and lead to various psychological effects and physical complications. Therefore, it is crucial to conduct further studies about childhood obesity and to aid policymakers in tackling the issue. Moreover, it is concluded that data on micronutrient deficiencies in the UAE population is scarce; therefore; it is important to conduct further studies aimed at assessing the micronutrient status of the UAE population.

## Figures and Tables

**Table 1:** Energy Malnutrition (stunting, wasting, underweight).

	Year	Title	Settings	Sample Size	Author
1	2019	Malnutrition-Inflammation Score VS Phase Angle in the Era of GLIM Criteria: A Cross-Sectional Study among Hemodialysis Patients in UAE	Hospital-based HD unit in the UAE.	70 hemodialysis patients.	Karavetian et al [46].
2	2020	Identification of Malnourished Patients using the Global Leadership Initiative on Malnutrition (GLIM) Diagnostic Criteria in the United Arab Emirates	Two hospitals in Dubai and Sharjah.	361 patients.	Karavetian et al [47].
3	2022	Nutritional Status and Adequacy of Feeding Practices in Infants and Toddlers 0–23.9 months living in the United Arab Emirates (UAE): Findings from the Feeding Infants and Toddlers Study (FITS) 2020	Abu Dhabi, Dubai, and Sharjah.	276 infants and toddlers aged 0–23.9 months.	Cheikh Ismail et al [43].
4	2022	Total Usual Nutrient Intakes and Nutritional Status of United Arab Emirates Children (<4 Years): Findings from the Feeding Infants and Toddlers Study (FITS) 2021	Abu Dhabi, Dubai, and Sharjah.	525 children aged 0–47.9 months	Nasreddine et al [48].

**Table 4:** Micronutrient deficiency.

	Year	Title	Settings	Sample Size	Author
1	2014	Vitamin D Status and Its Relationship with Metabolic Markers in Persons with Obesity and Type 2 Diabetes in the UAE: A Cross-Sectional Study.	Rashid Center for Diabetes and Research, a tertiary diabetes care facility in Ajman, UAE	309 men and women were randomly selected	Sadiya et al. [83]
2	2014	PP281-MON: Outstanding abstract: A Randomized Controlled Double-Blinded Clinical Trial of Vitamin D3 Supplementation on Obesity Parameters in Obese Type 2 Diabetes Subjects in the UAE.	Rashid Center for Diabetes and Research, a tertiary diabetes care facility in Ajman, UAE	87 vitamin D-deficient obese, type 2 diabetic participants.	Sadiya [84]
3	2019	Iron Deficiency Anemia in Infants of Hatta Suburb-UAE	Child health family clinic, Hatta Hospital-UAE	259 infants	Dileep et al. [86]
4	2020	Prevalence of overweight/obesity, anemia, and their associations among female university students in Dubai, United Arab Emirates: A cross-sectional study.	National University in Dubai, UAE	300 university students	Al Sabbah [58]
5	2020	Hospital-Based Prevalence of Iron Deficiency Anemia among Pre-School Children in Dubai	Sulaiman Al-Habib Medical Group’s private tertiary care hospital in Dubai, UAE	1595 children	Faysal et al. [87]

## Data Availability

Not applicable.

## Supplementary Materials

The following supporting information can be downloaded at: https://www.mdpi.com/article/10.3390/nu15020363/s1. File S1: Ministry of health and prevention, UAE-2019. National Obesity Study Among School Age Children (5–17 years old).
