# Supplementary material for: Nutrition Situation Analysis in the UAE: A Review Study
Source: Nutrients. 2023 Jan 11;15(2):363. doi: 10.3390/nu15020363 (PMC9861891; doi:10.3390/nu15020363)
Supplement: Supplementary file 1 [file nutrients-15-00363-s001.zip › nutrients-2127043-supplementary.pdf]

UNITED ARAB EMIRATES
MINISTRY OF HEALTH & PREVENTION

الإمارات العربية المتحدة
وزارة الصحة و وقاية المجتمع

مركز الإحصاء والأبحاث

قسم الإحصاء

دارسة السمنة لطلبة المدارس بعمر (5 -17 سنة) في الامارات العربية المتحدة للعام الدارسي 2018م – 2019م

1 مايو 2020

تمهيد

تم جمع البيانات الخاصة بالدراسة من خلال الكشف الشامل لطلبة المدارس بالامارات العربية المتحدة وذلك بعمل الفحوصات والقياسات البدنية للطلبة خلال العام الدراسي 2018م - 2019م و تم الإستعانة بالخرائط الخاصة بالسمنة والمعتمدة من قبل منظمة الصحة العالمية وذلك من خلال 804171 طالب وطالبة (حكومي / وخاص) على مستوى الامارات العربية المتحدة وتم استبعاد عدد 26035 سجل وذلك بنسبة 3.24 % من اجملى الطلبة وذلك بين طلبة اقل من خمس سنوات او اكثر من 18 سنة او ادخال تاريخ الميلاد بطريقة خاطئة او الوزن او اكثر من 200 كيلو جرام او الطول اكثر من 210 سم وذلك على مستوى الدولة وكانت اجمالى نسبة السمنة 17.35 % على مستوى الدولة للعام الدراسي 2018 م -2019 م.، والجدول التالى يوضح ماسبب شرحة

تحليل نتائج السمنة للأمارات العربية المتحدة للعام الدراسي 2018 / 2019

البيان	العدد / النسبة
أجمالي الطلبة	804171
سجلات ادخال تاريخ الميلاد خطا	717
سجلات العمر اكثر من 18 سنة	3889
سجلات اقل من 5 سنوات	21329
سجلات الوزن اكثر من 200 كجم	52
سجلات الطول اكثر من 210 سم	48
أجمالي السجلات الغير دقيقة او المستبعدة من النتائج	26035
السجلات الدقيقة	778136
نسبة السجلات الغير دقيقة	3.24
أجمالي الطلبة المصابين بالسمنة على مستوى الامارة (Zscoure) اكثر من 2 او يساويها	135042
اجمالى نسبة السمنة بالدولة	17.35

<u>نسبة تمثيل الطلبة حسب الامارة المشمولين بالدراسة للعام الدراسي 2018 /2019</u>

الشكل التالى يوضح نسبة تمثل الطلبة لكل امارة ونلاحظ ان اعلى تمثل لامارة ابوظبى حيث مثلت نسبة 37 % من اجمالى الطلبة المشمولين للدراسة تليها امارة دبى بنسبة 30 % واقل نسبة امارة ام القيوين بنسبة 1% من اجمالى الطلبة

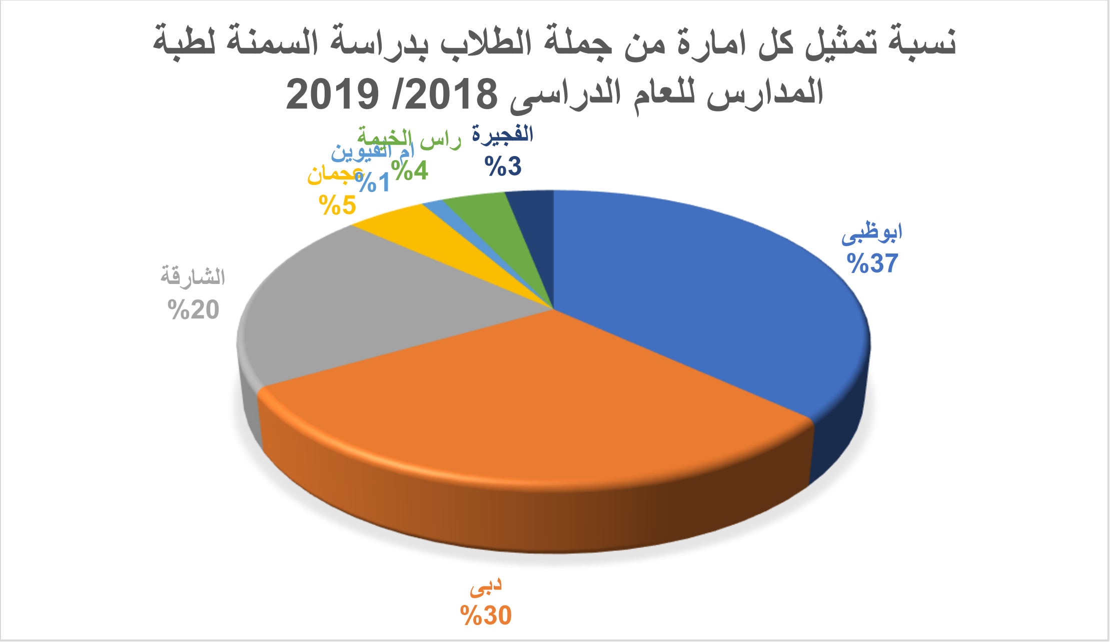

<u>بعض التعريفات الخاصة بالسمنة</u>

- الأطفال المستهدفون لمؤشر السمنة : هم الأشخاص في فئة العمر (5-17) سنة.

- السمنة : زيادة وزن الجسم عن حده الطبيعي نتيجة تراكم كميات زائدة من النسيج الدهني، على أن لا تكون هذه الزيادة نتيجة زيادة في العضلات كالرياضيين أو تراكم المياه بالجسم كالتورم والإستسقاء أو ضخامة في الهيكل العظمي . كذلك يعتبر الشخص مصابا بالسمنة إذا زادت "دلالة كتلة الجسم" عن 30كلغ / متر مربع.
- حساب كتلة الجسم للبالغين = الوزن بالكيلو جرام / (الطول بالمتر)2 . وتقسم درجات البدأنة بهذه الطريقة إلى :
  - (20-24.9) وزن طبيعي
  - (25-29.9) مرتفع
  - (30-34.9) سمنة من الدرجة الأولى
  - (35-39.9) سمنة من الدرجة الثانية
  - أكبر من 40 سمنة من الدرجة الثالثة (مفرطة)
- الأطفال في الفئة المستهدفة لمؤشر السمنة : الأطفال الذين يظهرون إنحرافا معيارياً أعلى من العلاقة الارتباطية بين متوسط الوزن و الطول بمقدار +2 فأعلى وفق الجداول والخرائط الخاصة والمعتمدة من منظمة الصحة العالمية في حساب السمنة لدى الأطفال .

التحليل الإحصائي

الجزء الأول على مستوى الدولة

وفيما يلى عرض لكل البيانات التي تم تحليلها على مستوى الدولة وحسب الامارة

أولا : اجمالى نسبة السمنة حسب الامارة للعام الدراسى 2018 /2019

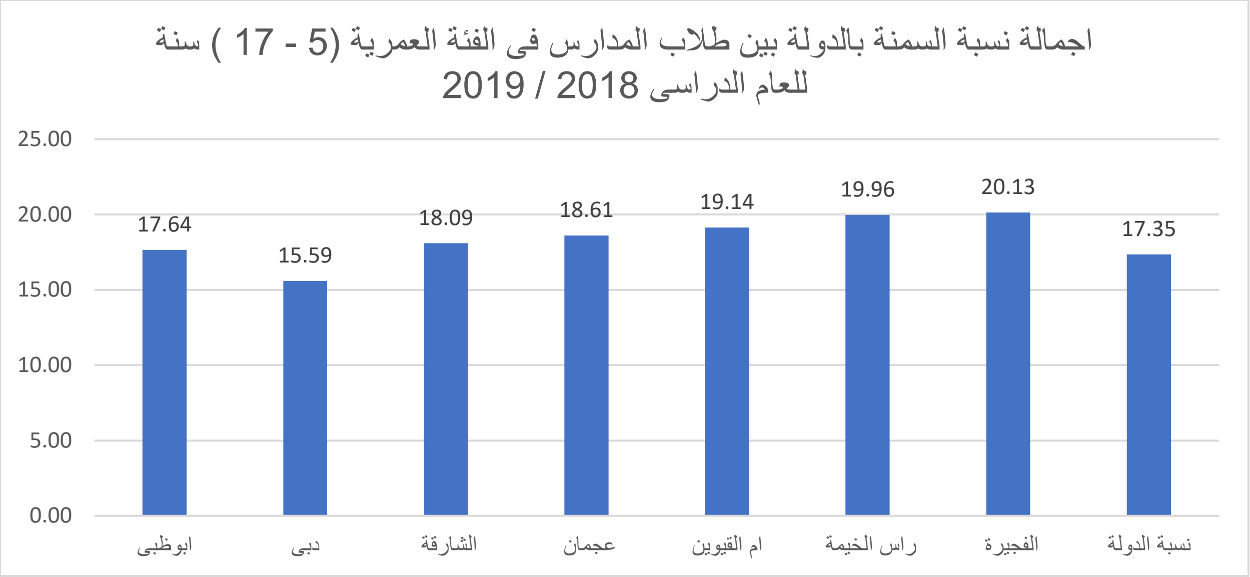

من الشكل السابق يتضح ان اجمالى نسبة السمنة بين الطلبة في الفئة العمرية من (5 _ 17) سنة للعام الدراسى 2018 / 2019 هو 17.35 % من اجمالى الطلبة وان اعلى نسبة إصابة بالسمنة بامارة الفجيرة حيث مثلت بنسبة 20.13 % واقل نسبة إصابة امارة دبى حيث مثلت بنسبة 15.59 % والجدول التالى يوضح البيانات حسب الامارة

اجمالى اعداد الطلبة المفحوصين والمصابين بالسمنة ونسبة السمنة حسب الامارة للعام الدراسي 2018/ 2019

البيان	ابوظبى	دبى	الشارقة	عجمان	ام القيوين	راس الخيمة	الفجيرة	الدولة
اجمالى الطلبة	287060	231326	156538	38725	10265	30674	23548	778136
المصابين بالسمنة	50639	36055	28312	7208	1965	6122	4741	135042
نسبة السمنة	17.64	15.59	18.09	18.61	19.14	19.96	20.13	17.35

<u>ثانيا : نسبة السمنة حسب الامارة والقطاع (حكومى / خاص) بين طلبة المدارس للعام الدراسي 2018 / 2019 فى فئة العمر (5 – 17) سنة</u>

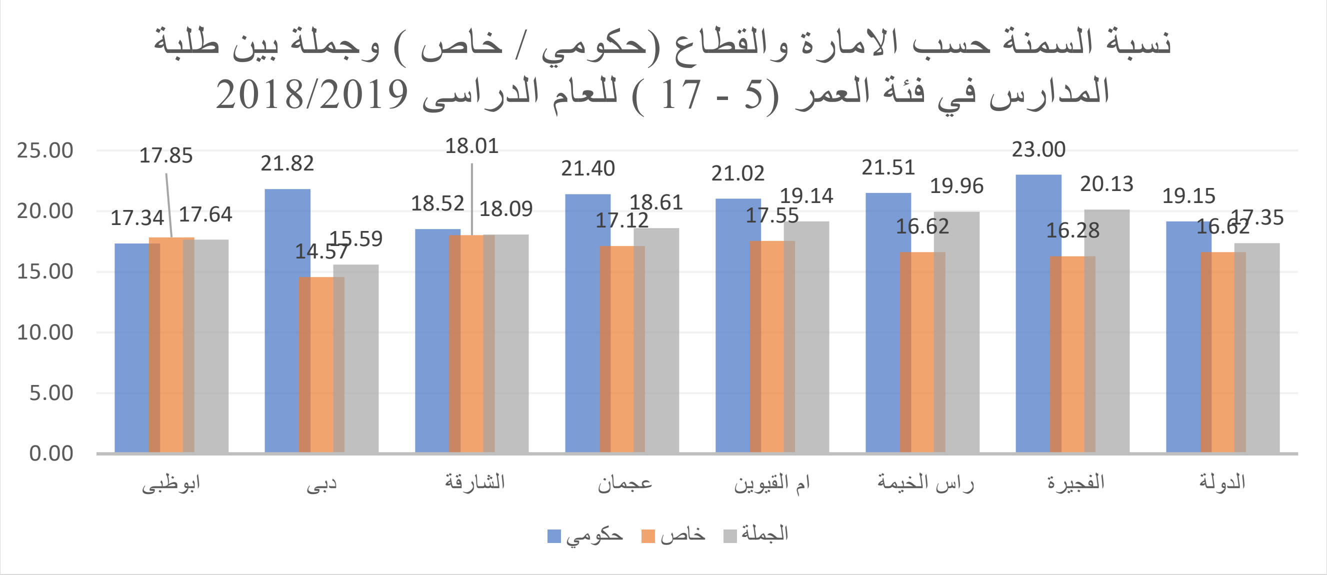

من الشكل السابق يتضح ان جملة السمنة بالدولة للقطاع الحكومى هو 19.15 % وان اجمالى نسبة السمنة بالقطاع الخاص هو 16.62 % وان اجمالى السمنة على مستوى الدولة هو 17.35 % ، وان اقل نسبة سمنة بالقطاع الحكومى بامارة ابوظبى وقد مثلت نسبة 17.34 % واعلى نسبة سمنة بالقطاع الحكومى بامارة الفجيرة وقد مثلت نسبة 23 % وكذلك اعلى نسبة إصابة بالقطاع الخاص بامارة الشارقة وقد مثلت نسبة 18.01 % واقل نسبة إصابة بالقطاع الخاص بامارة دبى وقد مثلت نسبة 14.57 % علما بان اجمالى نسبة الإصابة بالقطاع الخاص هو 16.62 % ، والجدول التالى يوضح البيانات حسب الامارة .

اعداد الطلبة حسب القطاع (حكومي / خاص) ونسبة السمنة والامارة للعام الدراسي 2018 /2019

البيان		ابوظبى	دبى	الشارقة	عجمان	ام القيوين	راس الخيمة	الفجيرة	الدولة
حكومي	اجمالى الطلبة	117238	32519	23198	13516	4705	20929	13508	225613
	المصابين بالسمنة	20330	7095	4297	2893	989	4502	3106	43212
	نسبة السمنة	17.34	21.82	18.52	21.40	21.02	21.51	22.99	19.15
خاص	اجمالى الطلبة	169822	198807	133340	25209	5560	9745	10040	552523
	المصابين بالسمنة	30309	28960	24015	4315	976	1620	1635	91830
	نسبة السمنة	17.85	14.57	18.01	17.12	17.55	16.62	16.28	16.62

الجزء الثانى : تحليل النتائج حسب الفئات العمرية على مستوى دبى والامارات الشمالية وذلك لعدم توافر بيانات امارة ابوظبى

- **اجمالى نسبة السمنة حسب الامارة لدبى والامارات الشمالية حيب الامارة بين طلاب المدارس في الفئة العمرية (5 – 17) سنة**

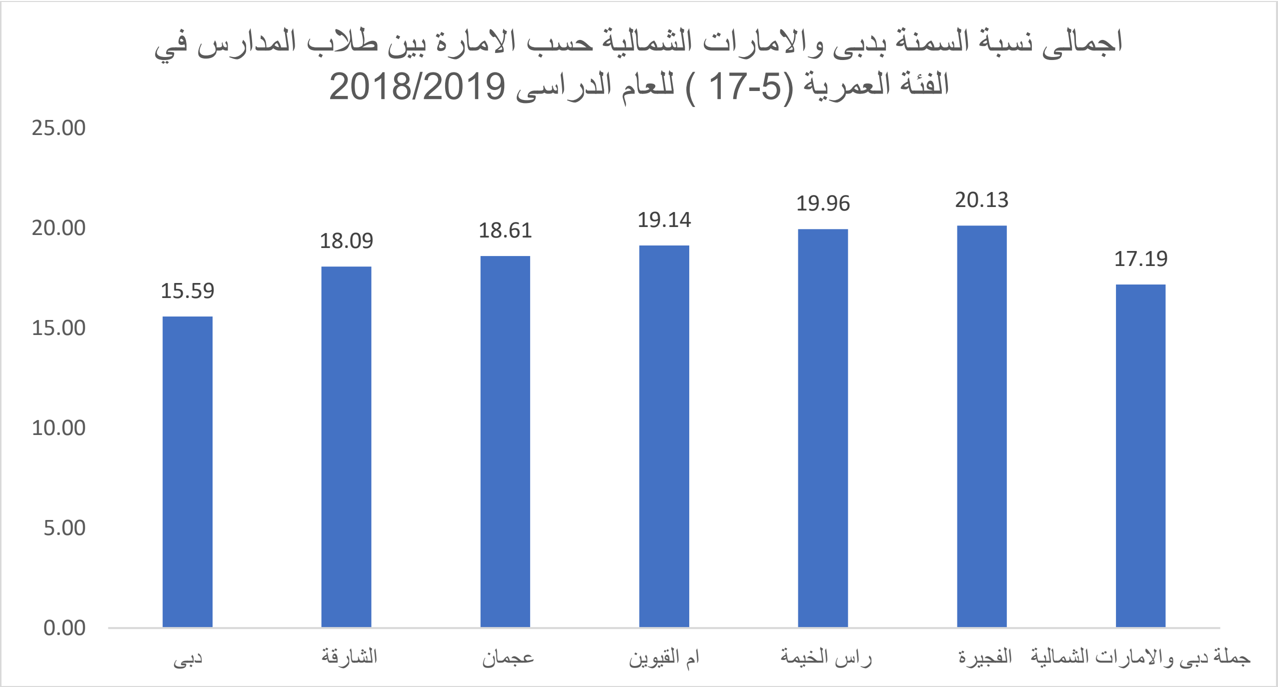

من الشكل السابق يتضح ان اجمالى نسبة السمنة بين الطلبة في الفئة العمرية من (5 _ 17) سنة بدبى والامارات الشمالية للعام الدراسى 2018 / 2019 هو 17.19 % من اجمالى الطلبة وان اعلى نسبة إصابة بالسمنة بامارة الفجيرة حيث مثلت بنسبة 20.13 % واقل نسبة إصابة امارة دبى حيث مثلت بنسبة 15.59 % .

إجمالي اعداد الطلبة المفحوصين والمصابين بالسمنة حسب الامارة بين طلبة المدارس للعام الدراسى (2018 / 2019) في الفئة العمرية (5 – 17) سنة

البيان	دبى	الشارقة	عجمان	ام القيوين	راس الخيمة	الفجيرة	دبى والامارات الشمالية
اجمالى الطلبة	231326	156538	38725	10265	30674	23548	491076
المصابين بالسمنة	36055	28312	7208	1965	6122	4741	84403
نسبة السمنة	15.59	18.09	18.61	19.14	19.96	20.13	17.19

- **<u>اجمالى نسبة السمنة في فئة العمر (5) سنوات حسب القطاع للعام الدراسى 2018 / 2019</u>**

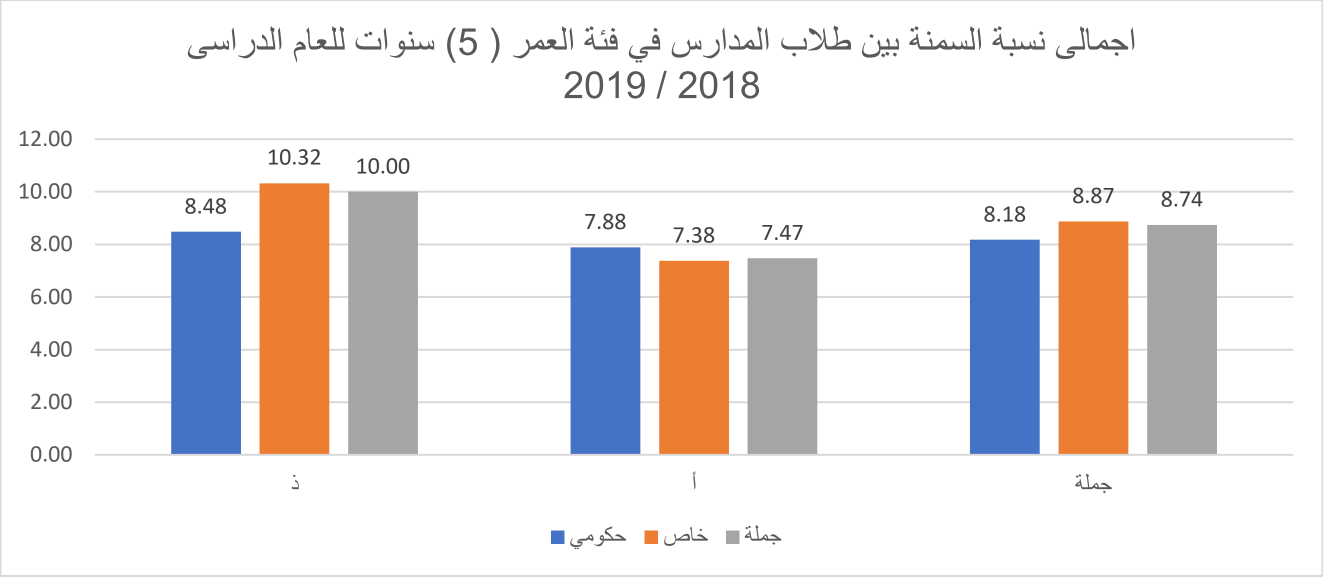

من الشكل السابق يتضح ان اجمالى نسبة بالقطاع الحكومى في فئة العمر (5) سنوات هي 8.18 % وبالقطاع الخاص هي 8.87 % في حين ان نسبة السمنة بالذكور في القطاع الخاص قد مثلت بنسبة 8.48% في حين مثلت بالقطاع الخاص نسبة 10.32 % ، اما بالنسبة للإناث فقد مثلت نسبة 7.88 % بالقطاع الحكومى ، ونسبة 7.38 % بالقطاع الخاص ، ومما سبق يتضح ان نسبة السمنة بالقطاع الخاص في هذه الفئة اعلى من مثيلاتها بالقطاع الحكومى

إجمالي اعداد الطلبة والمصابين بالسمنة ونسبة السمنة في الفئة العمرية (5) حسب القطاع للعام الدراسي 2019 / 2018

البيان	إجمالي الطلبة			الطلبة المصابين بالسمنة			قطاع حكومي		
	ذ	أ	جملة	ذ	أ	جملة	ذ	أ	جملة
حكومي	4080	4211	8291	346	332	678	8.48	7.88	8.18
خاص	19022	18521	37543	1964	1366	3330	10.32	7.38	8.87
جملة	23102	22732	45834	2310	1698	4008	10.00	7.47	8.74

❖ **نسبة السمنة حسب الامارة والقطاع (حكومى / خاص) بين طلاب المدارس في فئة العمر (5) سنوات للعام الدارسى 2019 / 2018**

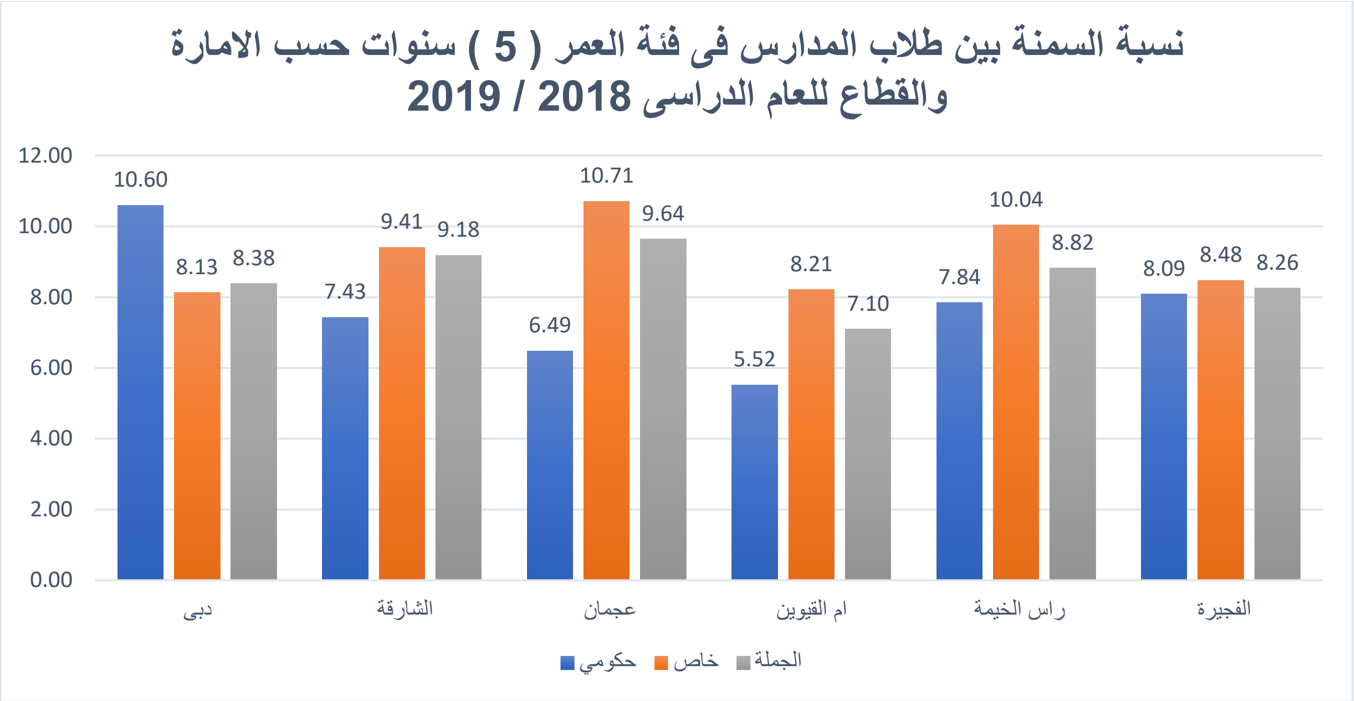

من الشكل السابق يتضح ان اعلى نسبة سمنة في فئة العمر (5) سنوات بالقطاع الحكومى بامارة دبى وقد مثلت بنسبة 10.6 % تليها امارة الفجيرة بنسبة 8.09 % واقل نسبة سمنة في هذه الفئة في امارة ام القيوين حسث مثلت نسبة 5.52 % ، اما القطاع الخاص فكانت اعلى نسبة بامارة عجمان وقد مثلت نسبة 10.71 % تليها امارة رأس الخيمة بنسبة 10.04 % واقل نسبة يامارة دبى بنسبة 8.13 % ،

إجمالي اعداد الطلبة والمصابين بالسمنة في فئة العمر (5) سنوات حسب القطاع (حكومي / خاص) ونسبة السمنة والامارة للعام الدراسي 2018/ 2019

البيان		دبى	الشارقة	عجمان	ام القيوين	راس الخيمة	الفجيرة	الجملة
حكومي	اجمالى الطلبة	2029	1845	956	489	1377	1595	8291
	المصابين بالسمنة	215	137	62	27	108	129	678
	نسبة السمنة	10.60	7.43	6.49	5.52	7.84	8.09	8.18
خاص	اجمالى الطلبة	17729	13960	2839	694	1106	1215	37543
	المصابين بالسمنة	1441	1314	304	57	111	103	3330
	نسبة السمنة	8.13	9.41	10.71	8.21	10.04	8.48	8.87

❖ **اجمالى نسبة السمنة فى فئة العمر (6 – 11) سنة حسب القطاع للعام الدراسى 2018 / 2019 م**

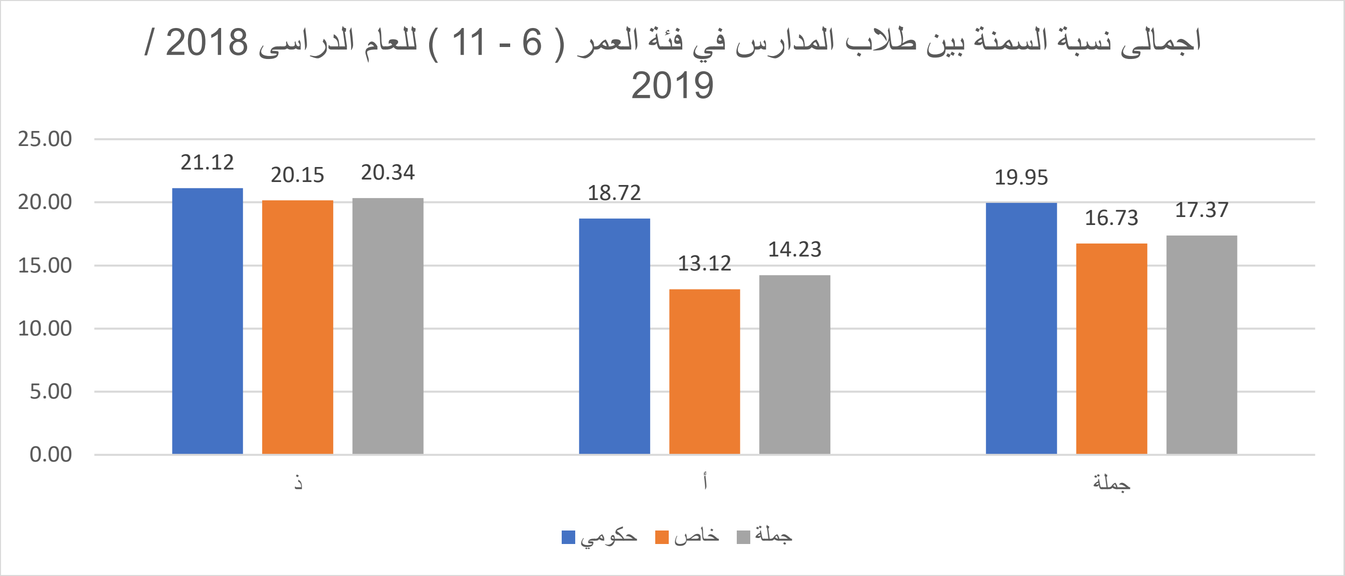

من الشكل السابق يتضح ان اجمالى نسبة السمنة بالقطاع الحكومى في فئة العمر (6 – 11) سنة هي 19.95 % وبالقطاع الخاص هي 16.73 % في حين ان نسبة السمنة بالذكور في القطاع الخاص قد مثلت بنسبة 20.15 % في حين مثلت بالقطاع االحكومى نسبة 21.12 % ، اما بالنسبة للإناث فقد مثلت نسبة 18.72 % بالقطاع الحكومى ، ونسبة 13.12 % بالقطاع الخاص ، ومما سبق يتضح ان نسبة السمنة بالقطاع الخاص وقد مثلت 16.73 % وهى اقل من القطاع الحكومى حيث مثل نسبة 19.95 %

اجمالى اعداد الطلبة والمصابين بالسمنة ونسبة السمنة في الفئة العمرية (6 – 11) حسب القطاع للعام الدراسى 2018 / 2019

البيان	اجمالى الطلبة			الطلبة المصابين بالسمنة			نسبة السمنة		
	ذ	أ	جملة	ذ	أ	جملة	ذ	أ	جملة
حكومي	27924	26621	54545	5898	4983	10881	21.12	18.72	19.95
خاص	113837	107698	221535	22937	14134	37071	20.15	13.12	16.73
جملة	141761	134319	276080	28835	19117	47952	20.34	14.23	17.37

❖ **نسبة السمنة حسب الامارة والقطاع (حكومى / خاص) بين طلاب المدارس فى فئة العمر (6 – 11) سنة للعام الدارسى 2018 / 2019**

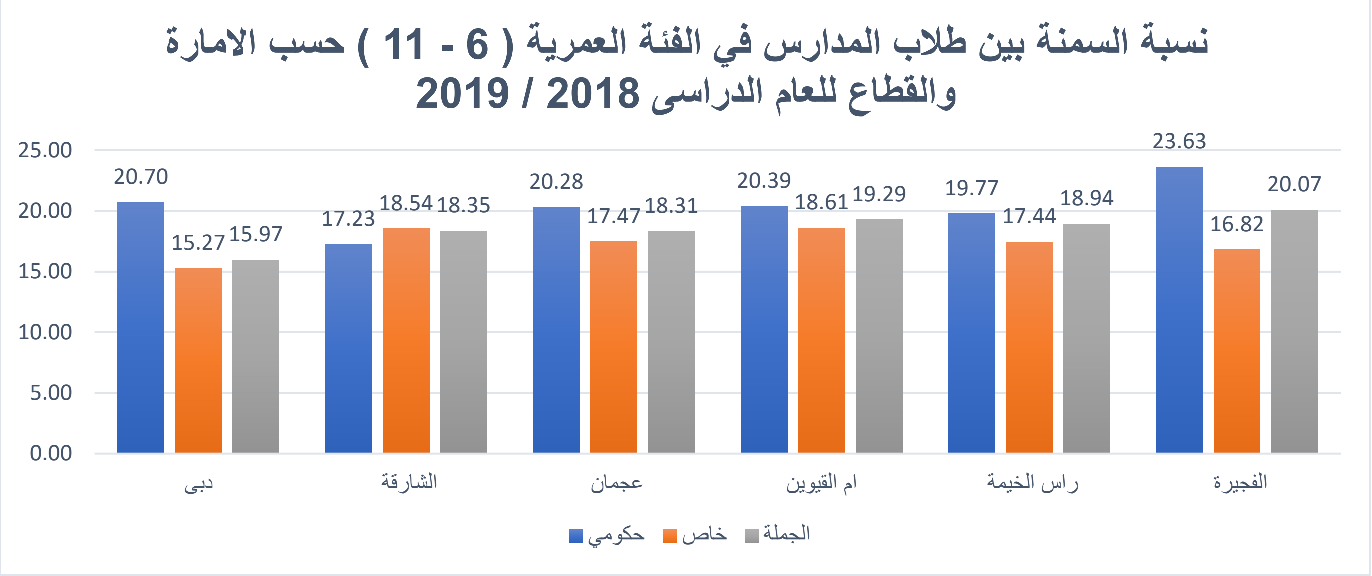

من الشكل السابق يتضح ان أعلى نسبة سمنة في فئة العمر (6 -11) سنة بالقطاع الحكومى بامارة الفجيرة فقد مثلت نسبة 23.63 % تليها امارة دبى بنسبة 20.70 % وان اقل نسبة سمنة فى هذه الفئة بامارة الشارقة وقد مثلت نسبة 17.23 % ، في حين ان اعلى نسبة سمنة بالقطاع الخاص في امارة ام القيوين وقد مثلت نسبة 18.61 % تليها امارة الشارقة وقد مثلت نسبة 18.54 % ، في ان اقل نسبة سمنة بالقطاع الخاص بامارة دبى وقد مثلت نسبة 15.27 % .

إجمالي اعداد الطلبة والمصابين بالسمنة في فئة العمر (6 - 11) سنة حسب القطاع (حكومي / خاص) ونسبة السمنة والامارة للعام الدراسي 2018/ 2019

البيان		دبى	الشارقة	عجمان	ام القيوين	راس الخيمة	الفجيرة	الجملة
حكومي	اجمالى الطلبة	16641	13237	6705	2099	9863	6000	54545
	المصابين بالسمنة	3444	2281	1360	428	1950	1418	10881
	نسبة السمنة	20.70	17.23	20.28	20.39	19.77	23.63	19.95
خاص	اجمالى الطلبة	111999	78517	15595	3418	5453	6553	221535
	المصابين بالسمنة	17103	14555	2724	636	951	1102	37071
	نسبة السمنة	15.27	18.54	17.47	18.61	17.44	16.82	16.73

<u>**اجمالى نسبة السمنة فى فئة العمر (12 – 14) سنة حسب القطاع للعام الدراسى 2018 / 2019 م**</u>

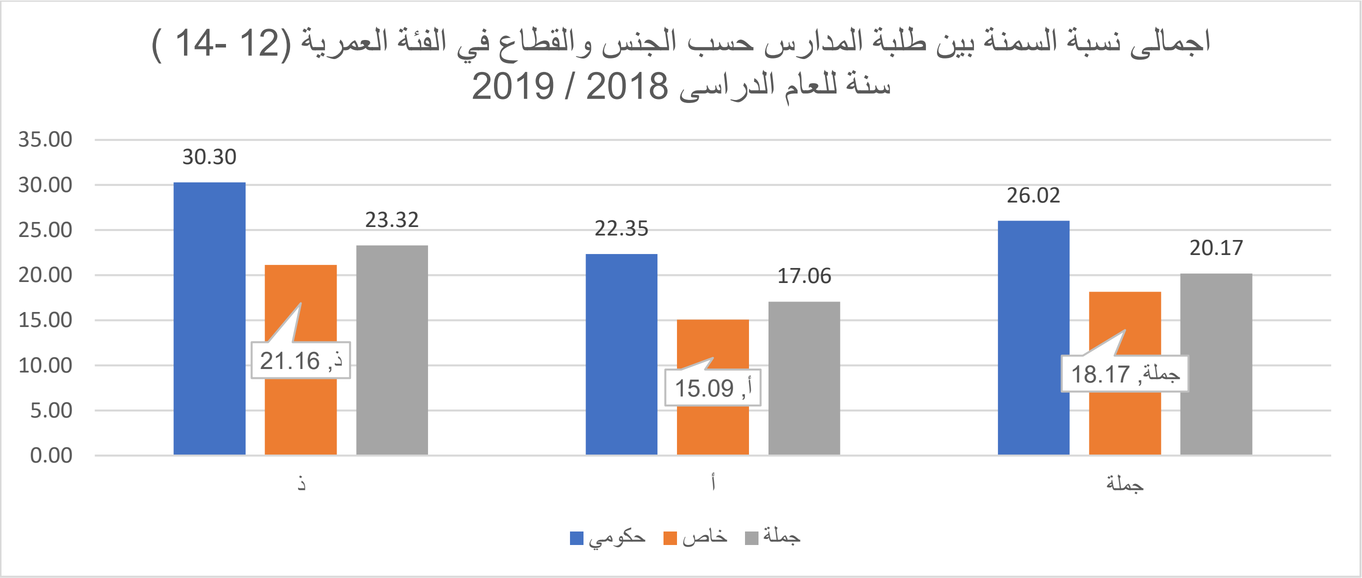

من الشكل السابق يتضح ان اجمالى نسبة السمنة بالقطاع الحكومى في فئة العمر (12 – 14) سنة هي 26.02 % وبالقطاع الخاص هي 18.17% في حين ان نسبة السمنة بالذكور في القطاع الخاص قد مثلت بنسبة 21.16 % في حين مثلت بالقطاع االحكومى نسبة 30.3 % ، اما بالنسبة للإناث فقد مثلت نسبة 22.35 % بالقطاع الحكومى ، ونسبة 15.09 % بالقطاع الخاص ، وان اجمالى نسبة السمنة بالقطاع الخاص اقل من اجمالى نسبة السمنة بالقطاع الحكومة ، والجدول التالى يوضح

اجمالى اعداد الطلبة والمصابين بالسمنة ونسبة السمنة في الفئة العمرية (12 – 14) حسب القطاع للعام الدراسى 2018 / 2019

البيان	اجمالى الطلبة			الطلبة المصابين بالسمنة			قطاع حكومي		
	ذ	أ	جملة	ذ	أ	جملة	ذ	أ	جملة
حكومي	11807	13769	25576	3578	3077	6655	30.30	22.35	26.02
خاص	38175	36918	75093	8077	5571	13648	21.16	15.09	18.17
جملة	49982	50687	100669	11655	8648	20303	23.32	17.06	20.17

نسبة السمنة حسب الامارة والقطاع (حكومى / خاص) بين طلاب المدارس فى فئة العمر (12 – 14) سنة للعام الدارسى 2018 / 2019

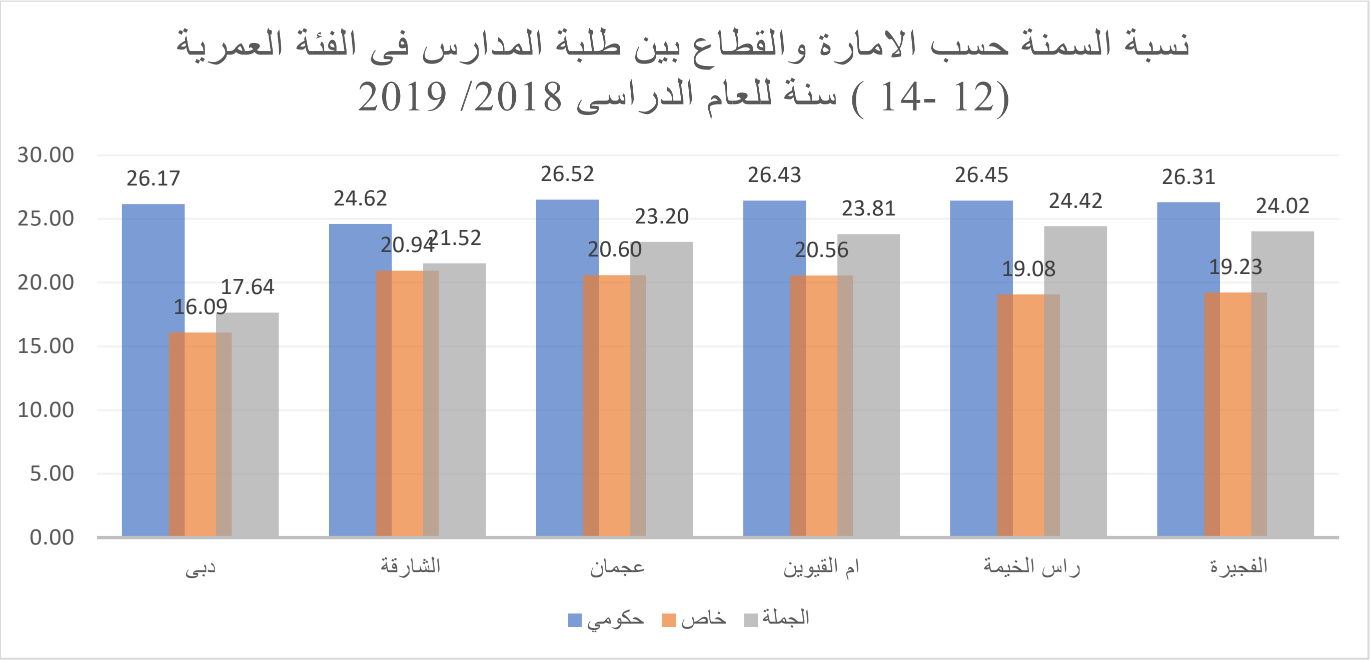

من الشكل السابق يتضح ان أعلى نسبة سمنة في فئة العمر (12 -14) سنة بالقطاع الحكومى بامارة عجمان فقد مثلت نسبة 26.52 % تليها امارة راس الخيمة بنسبة 26.43% ، وان اقل نسبة سمنة فى هذه الفئة بامارة الشارقة وقد مثلت نسبة 24.62 % ، في حين ان اعلى نسبة سمنة بالقطاع الخاص في امارة الشارقة وقد مثلت نسبة 20.94 % ، تليها امارة عجمان وقد مثلت نسبة 20.6% ، و ان اقل نسبة سمنة بالقطاع الخاص بامارة دبى وقد مثلت نسبة 16.09 %

إجمالي اعداد الطلبة والمصابين بالسمنة في فئة العمر (12 - 14) سنة حسب القطاع (حكومي / خاص) ونسبة السمنة والامارة للعام الدراسي 2018/ 2019

	البيان	دبي	الشارقة	عجمان	ام القيوين	راس الخيمة	الفجيرة	الجملة
حكومي	اجمالى الطلبة	7481	4696	3356	1188	5373	3482	25576
	المصابين بالسمنة	1958	1156	890	314	1421	916	6655
	نسبة السمنة	26.17	24.62	26.52	26.43	26.45	26.31	26.02
خاص	اجمالى الطلبة	41059	25111	4272	958	2034	1659	75093
	المصابين بالسمنة	6606	5258	880	197	388	319	13648
	نسبة السمنة	16.09	20.94	20.60	20.56	19.08	19.23	18.17

اجمالى نسبة السمنة فى فئة العمر (15 – 17) سنة حسب القطاع للعام الدراسى 2018 / 2019 م

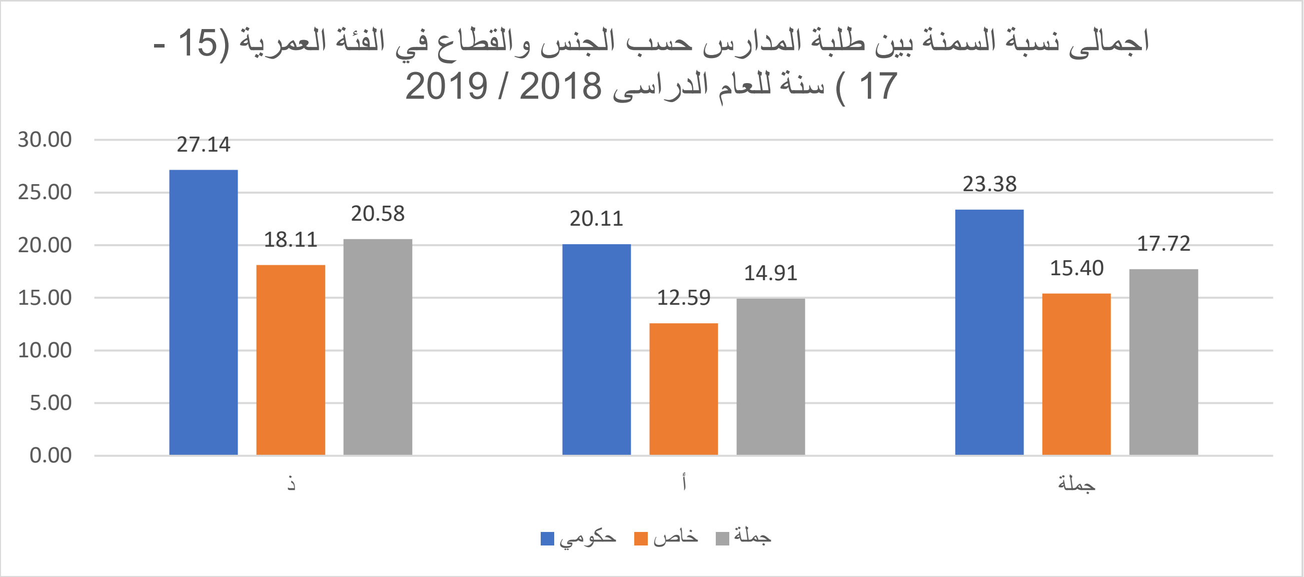

من الشكل السابق يتضح ان اجمالى نسبة السمنة بالقطاع الحكومى في فئة العمر (15 – 17) سنة هي 23.38 % وبالقطاع الخاص هي 15.4 % في حين ان نسبة السمنة بالذكور في القطاع الخاص قد مثلت بنسبة 18.11 % وقد مثلت بالقطاع االحكومى نسبة 27.14 % ، اما بالنسبة للإناث فقد مثلت نسبة 20.11 % بالقطاع الحكومى ، ونسبة 12.59 % بالقطاع الخاص ، وان اجمالى نسبة السمنة بالقطاع الخاص اقل من اجمالى نسبة السمنة بالقطاع الحكومة ، والجدول التالى يوضح .

اجمالى اعداد الطلبة والمصابين بالسمنة ونسبة السمنة في الفئة العمرية (15 – 17) حسب القطاع للعام الدراسى 2018 / 2019

البيان	اجمالى الطلبة			الطلبة المصابين بالسمنة			نسبة السمنة		
	ذ	أ	جملة	ذ	أ	جملة	ذ	أ	جملة
حكومي	9304	10659	19963	2525	2143	4668	27.14	20.11	23.38
خاص	24682	23848	48530	4469	3003	7472	18.11	12.59	15.40
جملة	33986	34507	68493	6994	5146	12140	20.58	14.91	17.72

نسبة السمنة حسب الامارة والقطاع (حكومى / خاص) بين طلاب المدارس فى فئة العمر (15 – 17) سنة للعام الدارسى 2018 / 2019

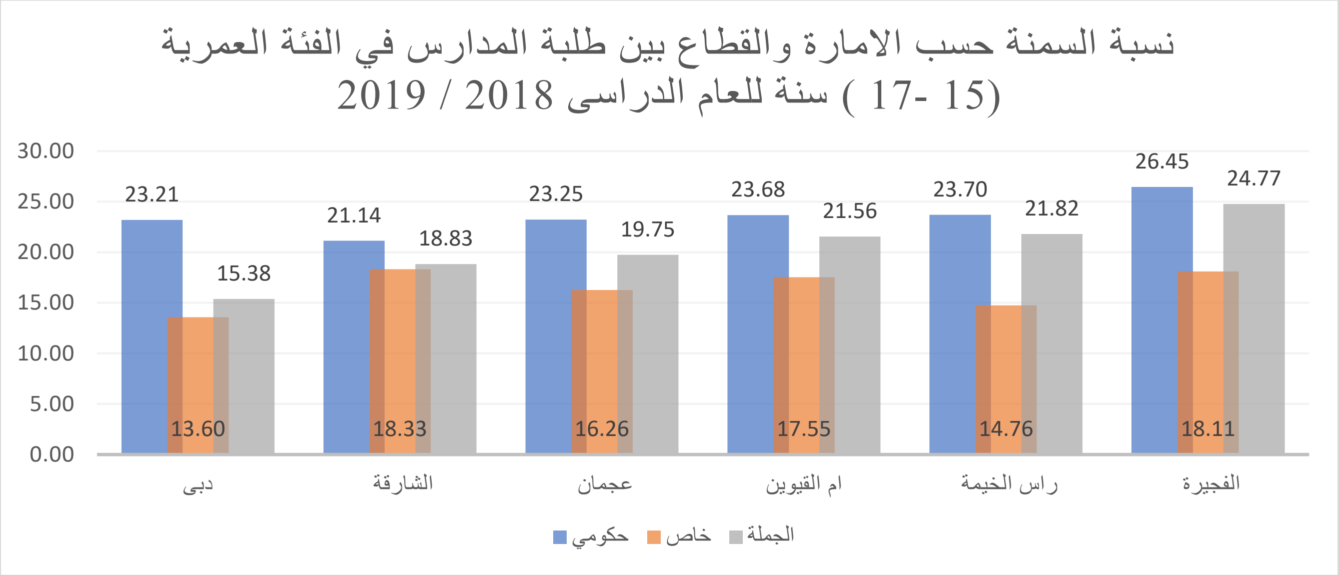

من الشكل السابق يتضح ان أعلى نسبة سمنة في فئة العمر (15 -17) سنة بالقطاع الحكومى بامارة الفجيرة فقد مثلت نسبة 26.45 % تليها امارة راس الخيمة بنسبة 23.7% ، واقل نسبة سمنة فى هذه الفئة بامارة الشارقة وقد مثلت نسبة 21.14 % ، في حين ان اعلى نسبة سمنة بالقطاع الخاص في امارة الشارقة وقد مثلت نسبة 18.33 % تليها امارة الفجيرة وقد مثلت نسبة 18.11 % ، و ان اقل نسبة سمنة بالقطاع الخاص بامارة دبى وقد مثلت نسبة 13.6 %

إجمالي اعداد الطلبة والمصابين بالسمنة في فئة العمر (12 - 14) سنة حسب القطاع (حكومي / خاص) ونسبة السمنة والامارة للعام الدراسي 2018/ 2019

البيان		دبى	الشارقة	عجمان	ام القيوين	راس الخيمة	الفجيرة	الجملة
حكومي	اجمالى الطلبة	6368	3420	2499	929	4316	2431	19963
	المصابين بالسمنة	1478	723	581	220	1023	643	4668
	نسبة السمنة	23.21	21.14	23.25	23.68	23.70	26.45	23.38
خاص	اجمالى الطلبة	28020	15752	2503	490	1152	613	48530
	المصابين بالسمنة	3810	2888	407	86	170	111	7472
	نسبة السمنة	13.60	18.33	16.26	17.55	14.76	18.11	15.40

تطور نسبة السمنة حسب الفئة العمرية والجنس والقطاع على مستوى دبى والامارات الشمالية للعام الدراسى 2018 / 2019

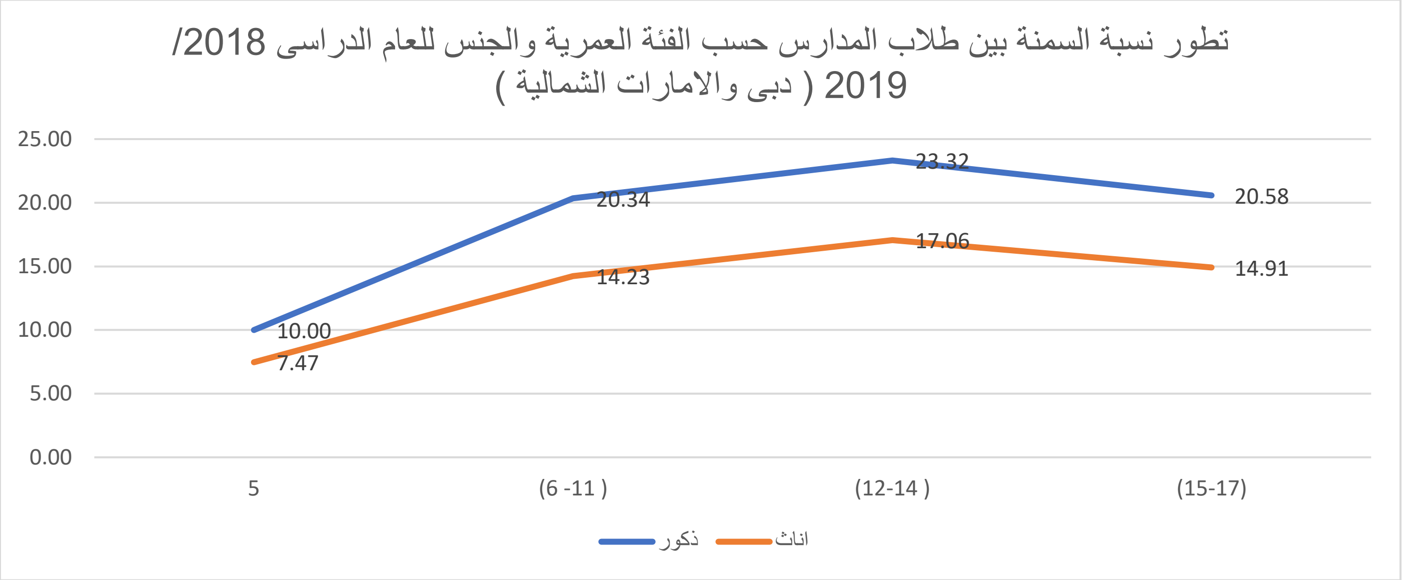

من الشكل السابق يتضح ان السمنة تبدا بالارتفاع في فئة العمر (6 – 11) وتصل الى اعلى معدلاتها في الفئة العمرية (12 – 14) سنة سواء بالاناث او الذكور وتعود للانخفاض التدريجى في الفئة العمرية (15 – 17) بنفس المعدلات تقريبا

تطور نسبة السمنة بين طلاب المدارس حسب الفئة العمرية والجنس للعام الدراسي 2018 / 2019

فئة العمر	5	(6 -11)	(12-14)	(15-17)
ذكور	10.00	20.34	23.32	20.58
اناث	7.47	14.23	17.06	14.91
جملة	8.74	17.37	20.17	17.72

نسبة السمنة حسب الجنسية والجنس بين طلبة المدارس في الفئة العمرية (5 –17) للعام الدراسي 2018 / 2019

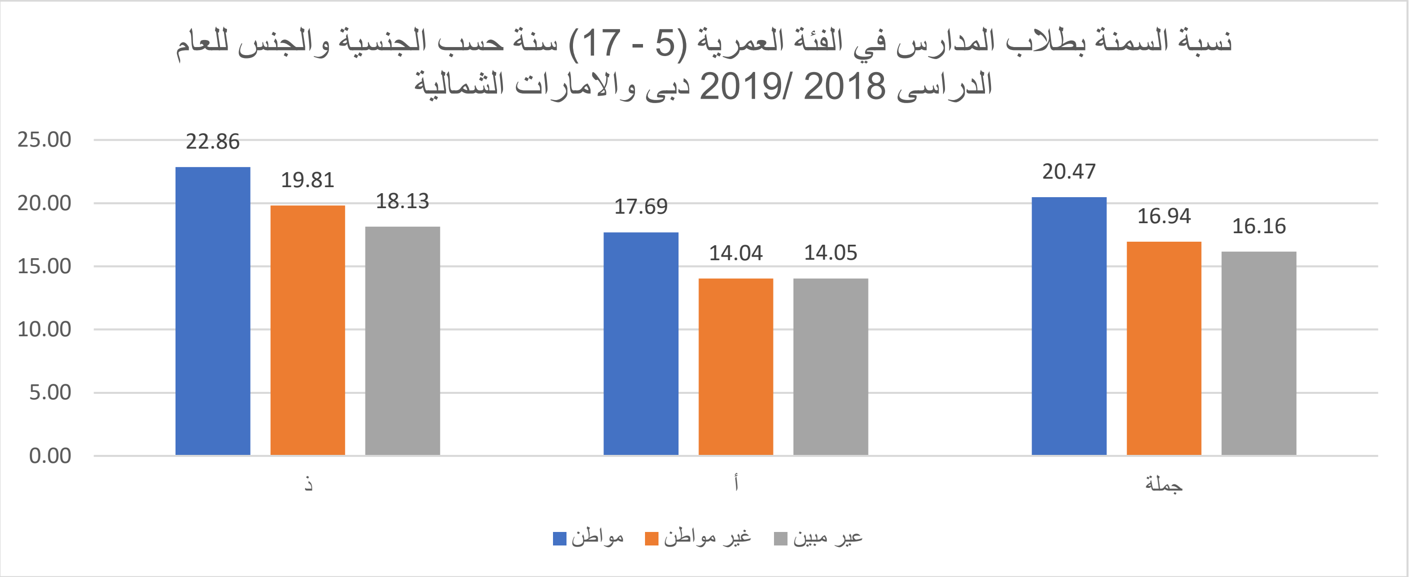

من الشكل السابق يتضح ان اعلى نسبة سمنة تتمثل بالذكور المواطنين وتمثل نسبة 22.86 % يليها الذكور الغير مواطنين بنسبة 19.81 % وان اقل نسبة سمنة بالاناث الغير مواطنين وتمثل نسبة 14.04 % في حين ان اجمالى نسبة السمنة لدى الاناث تمثل 16.94 % وهى اقل من اجمالى نسبةالسمنة بالذكور والتي مثلت 20.47 % ، مع العلم انه يوجد نسبة ممثلة في غير مبين الجنسية وقد تم استبعادها في التحليل ولكنا تظهر بالشكل حيث انه لايتضح ان هذه السجلات اذا كانت تمث مواطنين او غير مواطنين

نسبة السمنة حسب االجنسية والجنس في الفئة العمرية (5 - 17) بطلاب المدارس للعام الدراسى 2018 /2019 دبى والامارات الشمالية

البيان	اجمالى الطلبة			الطلبة المصابين بالسمنة			نسبة السمنة		
	ذ	أ	جملة	ذ	أ	جملة	ذ	أ	جملة
مواطن	19213	16524	35737	4392	2923	7315	22.86	17.69	20.47
غير مواطن	225157	221549	446706	44593	31100	75693	19.81	14.04	16.94
عير مبين	4461	4172	8633	809	586	1395	18.13	14.05	16.16
جملة	248831	242245	491076	49794	34609	84403	20.01	14.29	17.19

نسبة السمنة حسب الامارة والجنسية بين طلاب المدارس في فئة العمر (5 – 17) للعام الدراسي 2018 / 2019

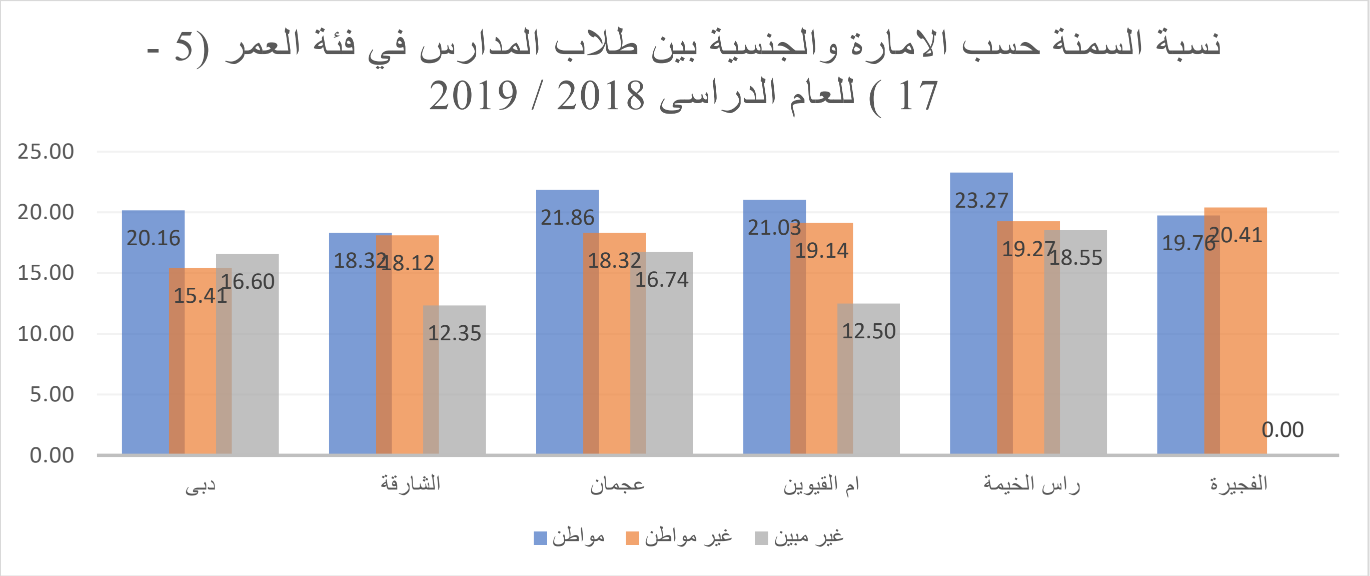

من الشكل السابق يتضح ان اعلى نسبة سمنة بالمواطنين بامارة راس الخيمة وقد مثلت نسبة 23.27 % ، واقل نسبة سمنة بالمواطنين بامارة الشارقة وقدمثلت نسبة 18.32 % ، وان اعلى نسبة سمنة بالغير مواطنين بامارة الفجيرة وقد مثلت نسبة 20.41 % واقل نسبة سمنة بامارة دبى وقد مثلت نسبة 15.41 % ، علما بان هذه النسب قد تتغير في حالة تعديل الادخال الخاص بفئة الغير مبين مثل ماهو موجود بالفعل بامارةالفجيرة حيث لايوجد حالات غير مبية الجنسية وهذه النسب على ماهة وارد بالسجلات التي تم عليها التحليل

إجمالي اعداد الطلبة ونسبة السمنة حسب الامارة والجنسية بين طلاب المدارس في الفئة العمرية (5-17) للعام الدراسى 2018 / 2019

	البيان	دبي	الشارقة	عجمان	ام القيوين	راس الخيمة	الفجيرة	الجملة
مواطن	اجمالى الطلبة	8044	6272	5531	523	5380	9987	35737
	المصابين بالسمنة	1622	1149	1209	110	1252	1973	7315
	نسبة السمنة	20.16	18.32	21.86	21.03	23.27	19.76	20.47
غير مواطن	اجمالى الطلبة	221607	149108	28069	9590	24771	13561	446706
	المصابين بالسمنة	34155	27020	5141	1836	4773	2768	75693
	نسبة السمنة	15.41	18.12	18.32	19.14	19.27	20.41	16.94
غير مبين	اجمالى الطلبة	1675	1158	5125	152	523	0	8633
	المصابين بالسمنة	278	143	858	19	97	0	1395
	نسبة السمنة	16.60	12.35	16.74	12.50	18.55	0.00	16.16

الخلاصة :

1. نسبة السمنة دائما بالذكور اعلى من الاناث في جميع الفئات العمرية
2. نسبة السمنة بالطلبة المواطنين اعلى من الغير مواطنين
3. اقل نسبة سمنة بامارة دبى وقد مثلت 15.59%
4. قل نسبة سمنة بالقطاع الخاص بامارة دبى وقد مثلت نسبة 14.57 %اقل نسبة سمنة بفئة العمر 5 سنوات بالقطاع الحكومى بامارة ام القيوين وقد مثلت 5.52 %
5. اعلى نسبة سمنة بفئة العمر 5 بالقطاع الخاص بامارة عجمان وقد مثلت 10.71 %
6. امارة ابوظبى هي الامارة الوحيدة التي تقل بها نسبة السمنة بين طلاب القطاع الحكومى عن القطاع الخاص بخلاف باقى الامارات الأخرى
7. نسبة السمنة تبدا بالارتفاع التدريجى بداية من عمر 5 سنوات حتى تصل الى الذرة في عمر (12 – 14) ثم تبدا بالانخفاض في فئة العمر (15-17) وهذا ينطبق على جميع الامارات بدون استثناء
8. نسبةا لسمنة في جميع الفئات العمرية بالذكور اعلى من الاناث

التوصيات :

1. عمل ورش تدريبية وتوعوية لكادر التمريض بالمدارس وذلك لرفع كفاءة الادخال للبيانات وتنمية الوعى الاحصائى لديهم لتوضيح مدى تاثر النتائج بما يمكن ان يقوما بإدخال البيانات بطريقة خطا وكذلك عرض نتائج الدراسة عليهم
2. اعداد وضبط النظام اللالكترونى المستخدم بما يتوائم مع المتطلبات والمخرجات المطلوبة لحساب نتائج السمنة بين طلاب المدارس
3. عمل حوافز تشجيعية للطاقم المدخل البيانات
4. عمل حوافز تشجيعية للطلبة الذين يمارسون نشاط بدنى
5. تطبيق برامج تدريبية بين طلبة المدارس بجانب الورش والندوات التثقيفية عن خطورة السمنة
